# PUM1 and PUM2 promote translation of chromatin regulators to ensure mammalian spermatogenesis

**DOI:** 10.1126/sciadv.aed5708

**Published:** 2026-07-17

**Authors:** Min Zang, Siyu Liu, Zhengyao Xie, Ding Yang, Ke Wang, Yan Ding, Tong Zhu, Shikun Zhang, Tingting Zhao, Bing Yao, Mingxi Liu, Kaibo Lin, Eugene Yujun Xu

**Affiliations:** ^1^ State Key Laboratory of Reproductive Medicine and offspring Health, Nanjing Medical University, Nanjing, Jiangsu, China, 211166.; ^2^ Department of Reproductive Medicine, Jinling Hospital, Affiliated Hospital of Medical School, Nanjing University, Nanjing, Jiangsu, China, 210002.; ^3^ Department of Assisted Reproduction, Shanghai Ninth People’s Hospital, Shanghai Jiao Tong University School of Medicine, Shanghai, China, 200021.; ^4^ State Key Laboratory of Reproductive Medicine and Offspring Health, The Affiliated Taizhou People’s Hospital of Nanjing Medical University, Taizhou School of Clinical Medicine, Nanjing Medical University, Nanjing, China, 211166.; ^5^ Cellular Screening Center, Biological Sciences Division, The University of Chicago, Chicago, USA, IL 60637.

## Abstract

Translational control is essential for male germ cell development, yet how post-transcriptional regulation is coupled to chromatin remodeling during spermatogenesis remains poorly understood. Here, we show that the RNA-binding proteins PUMILIO1 (PUM1) and PUMILIO2 (PUM2) promote translation of mRNAs encoding chromatin regulators in the mouse testis. Conditional deletion of *Pum1* and *Pum2* in germ cells at multiple developmental stages results in spermatogenic failure, defective nuclear shaping, impaired histone-to-protamine exchange, and complete male sterility. Polysome profiling and ribosome sequencing reveal selective reductions in translation efficiency of chromatin regulators, including histone modifiers and nucleosome remodelers, in *Pum1/2*-deficient testes. Mechanistically, PUM1/2 associate with DAZL and PABPC1 to form a germ cell–specific translational activation complex that enhances protein output with little impact on mRNA stability. Together, these findings redefine mammalian PUM proteins as context-dependent translational activators in the germline and uncover a post-transcriptional mechanism that links RNA regulation to chromatin dynamics during spermatogenesis.

## Teaser

PUM1/2 cooperate with DAZL and PABPC1 to selectively enhance translation of chromatin regulators required for spermatogenesis.

## INTRODUCTION

Spermatogenesis is a highly orchestrated developmental process that transforms diploid spermatogonia into haploid spermatozoa through mitotic proliferation, meiotic divisions, and spermiogenesis. These transitions are accompanied by extensive chromatin remodeling and dynamic epigenetic regulation, which underlie the unique gene expression programs required for germ cell differentiation (*1*–*5*). In particular, postmeiotic haploid spermatids undergo dramatic chromatin reorganization, characterized by histone modifications, transition protein incorporation, and histone-to-protamine transition, culminating in the highly compacted chromatin of mature sperm (*6*–*8*). Chromatin regulators such as BRDT, CTCF, SMARCA5, PRC2 components, and SIN3A play indispensable roles in these processes, yet the mechanisms that ensure their precise expression and timing remain incompletely understood (*9*–*16*).

A unique challenge in male germ cell development is that transcription is not continuous. Transcriptional activity peaks in spermatocytes and round spermatids but diminishes sharply during spermiogenesis as chromatin compaction progresses (*17*, *18*). Thus, germ cells rely heavily on post-transcriptional mechanisms, particularly translational control, to ensure the timely production of proteins during transcriptionally silent periods. RNA-binding proteins (RBPs) are central mediators of this regulation, coordinating mRNA stability, storage, and translation (*19*–*21*) Several RBPs, including DAZL, DDX20, FXR1, CEP112 and MIWI, have been shown to promote translation of mRNAs essential for spermatogenesis (*22*–*26*). Yet the RBPs that specifically control the translation of chromatin regulators—key drivers of meiotic progression and chromatin compaction—have remained unidentified.

The Pumilio (PUM) family of RBPs are evolutionarily conserved translational regulators from invertebrates to mammals. They recognize UGUA-containing motifs, termed PUMILIO Binding Elements (PBE), within the 3′UTRs of target mRNAs via their Pumilio Homology Domain (PUM-HD) (*27*, *28*). In Drosophila and *C. elegans*, Pumilio homologs are essential for germline stem cell maintenance, cell fate specification, and prevention of premature differentiation through translational repression of developmental regulators (*29*–*31*). Mammalian genomes encode two homologs, PUM1 and PUM2, which share high structural similarity and are broadly expressed. Genetic studies have implicated PUM1 and PUM2 in embryonic development, stem cell pluripotency, organ size control, and genome stability, with mutations associated with defects in proliferation, differentiation, and tissue homeostasis (*32*–*40*). In the germline, single knockout studies revealed roles for PUM1 or PUM2 in safeguarding spermatogenesis, yet with relatively mild or incompletely penetrant phenotypes, suggesting functional redundancy (*32*, *34*, *36*). Whether the stem cell–associated functions of Pumilio proteins observed in invertebrates (*19*, *29*, *41*) are conserved, modified, or redeployed during mammalian germline development has remained unresolved and requires analysis of combined *Pum1* and *Pum2* loss in vivo.

PUM proteins are generally viewed as translational repressors and mRNA decay factors, based on structural, biochemical, and transcriptome-wide studies showing PUM1/2 bind PBE in the 3’ UTR and recruit decay machinery (*41*–*44*). However, emerging evidence suggests that PUM function may be context-dependent. In specific cellular settings, including hematopoietic progenitors and neurons, PUM proteins have been reported to positively influence protein output from select targets, although the underlying mechanisms and physiological relevance remain poorly defined (*45*, *46*). Whether such non-canonical, translation-promoting functions operate in the mammalian germline—where translational control is essential due to transcriptional silencing—has not been addressed.

Here, we demonstrate that PUM1 and PUM2 function as translational activators during spermatogenesis. Using enhanced cross-linking immunoprecipitation (eCLIP), we identify chromatin regulator mRNAs as direct PUM1/2 targets. We show that PUM1/2 associate with the translational activators DAZL and PABPC1 to promote protein output with minimal effects on mRNA stability. Conditional deletion of *Pum1* and *Pum2* in germ cells at multiple developmental stages leads to defective meiotic progression, impaired chromatin remodeling, abnormal sperm morphology, and male sterility. Together, these findings support mammalian PUM proteins as context-dependent translational regulators and reveal a post-transcriptional mechanism that links RNA regulation to chromatin dynamics during spermatogenesis.

## RESULTS

### PUM1 is more abundant than PUM2 in spermatocytes and later germ cell stages

**Fig. 1.**
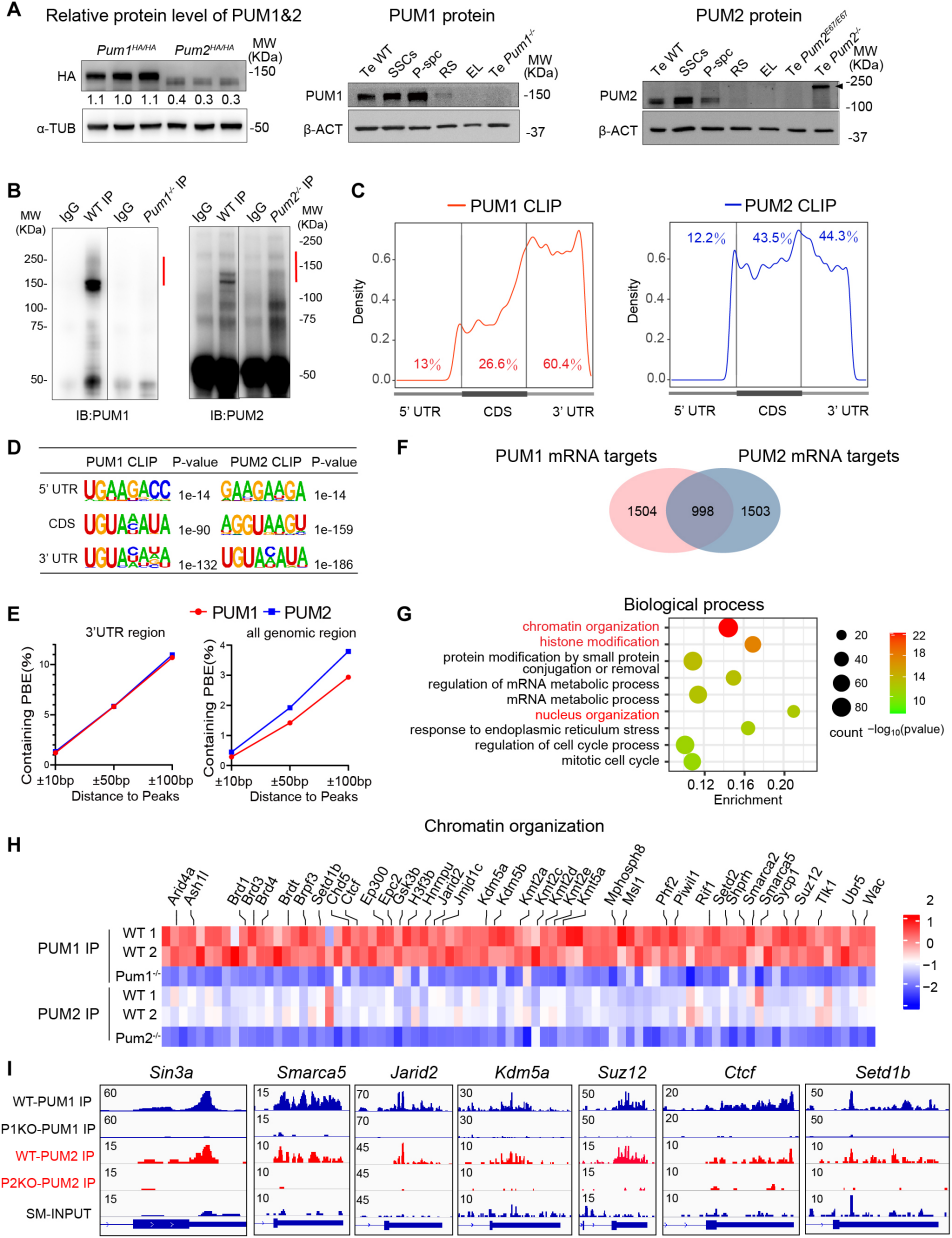
PUM1 is the predominant PUF protein in testes and co-targets chromatin regulator mRNAs with PUM2. (**A**) Protein abundance of PUM1 and PUM2 in testis and isolated germ cell populations via immunoblot analysis of testis lysates from 2-month-old *Pum1*-HA and *Pum2*-HA knock-in mice. Cultured spermatogonial stem cells (SSCs) and STA-PUT-isolated germ cell populations, including pachytene spermatocytes (P-Spc), round spermatids (RS), and elongating spermatids (EL), were used. (**B**) Immunoblot validation of PUM1 and PUM2 immunoprecipitation for eCLIP experiments. Protein-RNA complexes (marked by red lines) from WT and the corresponding knockout control testis lysates are shown, along with IgG controls. (**C**) Distribution of PUM1 and PUM2 eCLIP peaks across transcript regions. PUM1 binding is predominantly enriched in 3′UTRs, whereas PUM2 binds both 3′UTRs and coding sequences (CDS). (**D**) De novo motif analysis of PUM1 and PUM2 binding sites. PUM1 peaks contain canonical PUM-binding elements (PBEs; also referred to as PRE in prior literature). PUM2 peaks contain both canonical PBEs and distinct CDS-associated motifs. (**E**) Proportion of peaks containing the canonical PBE (UGUANAUA) within different distances from eCLIP peak centers for 3’UTR targets and for all PUM1/2 genomic targets. (**F**) Overlap of PUM1 and PUM2 eCLIP target mRNAs. Peaks with fold enrichment >8 and peak height > 3 were defined as high-confidence binding sites. (**G**) Gene Ontology (GO) analysis of mRNAs co-targeted by PUM1 and PUM2. The top 10 enriched biological processes are shown; chromatin organization and histone modification are the most significantly enriched (see also fig. S2E). (**H**) Heatmap of eCLIP peaks for chromatin regulators from top-enriched pathways. Peak heights were normalized by z-score and displayed in the heatmap, where z = (X - μ)/σ (X: original peak height; μ: mean of the dataset; σ: standard deviation). (**I**) Genome browser tracks showing 3′UTR binding of PUM1 and PUM2 on representative chromatin regulator mRNAs. P1KO, Pum1KO; P2KO, Pum2KO.

PUM1 and PUM2 are the two conserved vertebrate members of the PUF (PUMILIO and FBF) RNA-binding protein family. Despite their extensive evolutionary conservation, the relative expression and contributions of PUM1 versus PUM2 during spermatogenesis have remained unclear. To address this, we generated *Pum1-HA* and *Pum2-HA* knock-in mice (fig. S1, A to C), enabling direct comparison of protein levels with the same antibody. Immunohistochemistry revealed that both PUM1 and PUM2 are expressed from spermatogonia through spermatocytes, but with distinct dynamic profiles: PUM1 is enriched in spermatocytes, whereas PUM2 is highest in spermatogonial stem cells (SSCs) (fig. S1, D to F). Quantitative immunoblotting of purified spermatogenic populations demonstrated that overall testicular PUM1 levels are ∼2.5-fold higher than PUM2, with PUM1 peaking in pachytene spermatocytes and declining in round and elongating spermatids (Fig. 1A). In contrast, PUM2 is most abundant in SSCs and decreases progressively during meiosis (Fig. 1A). Thus, PUM1 is the predominant paralog in spermatocytes, while PUM2 is enriched earlier in SSCs. This asymmetric expression pattern suggests that PUM1 may play a predominant role during meiotic and postmeiotic stages of spermatogenesis.

### PUM1 and PUM2 co-target mRNAs encoding chromatin regulators in the testis

To elucidate the molecular functions of PUM proteins in spermatogenesis, we mapped their RNA targets in adult testes using enhanced CLIP (eCLIP) (Fig. 1B). Independent immunoprecipitations with PUM1 and PUM2 antibodies yielded highly reproducible binding profiles (R^2^ = 0.944 and 0.928 between replicates) (fig. S2A, Fig. 1B, and table S2). PUM1 peaks were enriched in 3′UTRs, while PUM2 showed broader occupancy in both CDS and 3′UTRs, with preference for first and last exons, resembling PUM1 and PUM2 binding profiles in somatic cells (Fig. 1C and fig. S2, B and C). Motif analysis identified the canonical PUM-binding element (UGUANAUA) in both regions for PUM1, whereas PUM2 recognized both the canonical motif in 3′UTRs and a distinct AGGUAAGU sequence in CDS (Fig. 1D), consistent with reported context-dependent binding preferences (*42*).

To further assess the relationship between canonical PUM-binding elements (PBEs) and PUM1/2 binding sites, we examined the positional overlap between PBEs and eCLIP peak centers. Only a small fraction of PUM1/2 peaks overlapped with PBEs within defined distances on 3′UTRs (∼1–10%, depending on the distance threshold), with even lower overlap observed when considering all genomic PUM targets (Fig. 1E).

Across replicates, PUM1 and PUM2 bound ∼2500 transcripts each, with 998 shared targets (Fig. 1F and fig. S2D). Gene ontology analysis revealed significant enrichment for chromatin organization and histone modification pathways, including components of histone acetyltransferase complexes and SWI/SNF remodeling machinery (Fig. 1, G and H and fig. S2, D and E). Other enriched categories included mRNA metabolism and cell cycle control, aligning with classical PUM family functions. Visualization of representative chromatin dynamics targets such as *Sin3a*, *Smarca5*, *Jarid2*, *Kdm5a*, *Suz12*, *Ctcf* and *Setd1b* confirmed robust peaks in 3′UTRs for both PUM1 and PUM2, absent in mutant controls (Fig. 1I and fig. S2G). Importantly, comparison with published PUM CLIP data from somatic cells revealed similar genomic distributions (fig. S2C) and enrichment for chromatin regulators (fig. S2H), indicating that key features of PUM binding logic are shared across cell types (*47*–*50*). RNA immunoprecipitation validated selected targets (fig. S2F).

Although PUM1/2 bind transcripts across diverse functional categories, chromatin regulators emerged as the most consistently enriched and conserved group across independent datasets. Together, these data establish chromatin regulators as one of the most prominent and conserved functional classes among shared PUM1/2 targets in the testis.

### Germ cell deletion of *Pum1* and *Pum2* results in male sterility

**Fig. 2.**
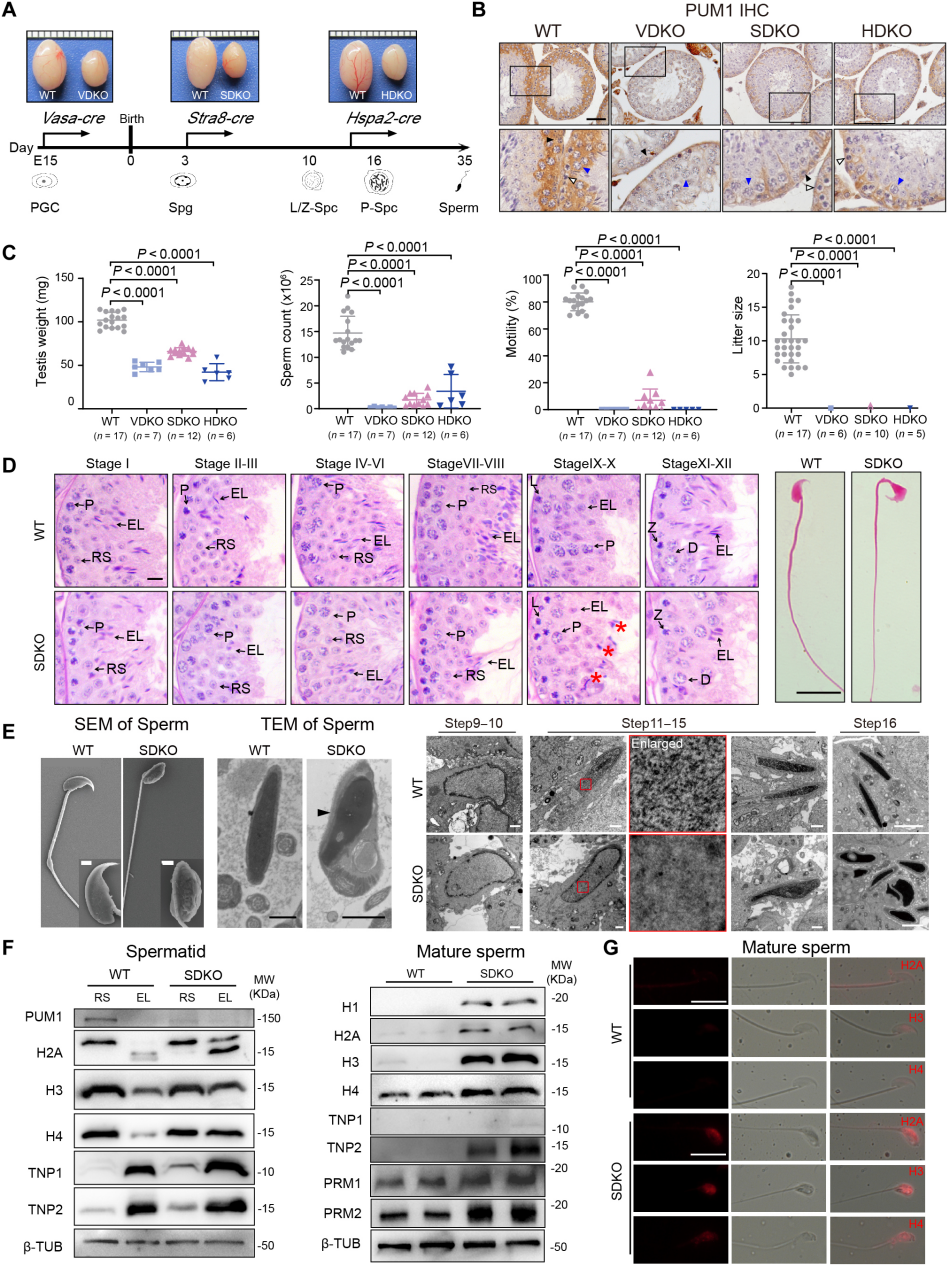
Germ cell-specific deletion of *Pum1/2* causes sterility and defective spermiogenesis. (**A**) Gross testis morphology of germ cell-specific conditional double knockout (cDKO) mice. Mice carrying Cre drivers initiated at distinct stages of germ cell development were analyzed. (**B**) Immunohistochemical (IHC) validation of *Pum1* depletion in cDKO testes. Efficient loss of PUM1 signal (brown) is observed in germ cells across developmental stages: spermatogonia (black arrowhead), leptotene/zygotene spermatocytes (white arrowhead), and pachytene/diplotene spermatocytes (blue arrowhead). Nuclei are counterstained with hematoxylin (blue color). Scale bar: 50 μm. (**C**) All three cDKO models display reduced testis size, oligozoospermia, and infertility. Statistical significance was determined by Student’s *t* test. (**D**) Histological analysis reveals defective spermiogenesis in SDKO testes. Hematoxylin and eosin (H&E) staining of seminiferous tubules from SDKO and control mice shows abnormal nuclear morphology and cytoplasmic vacuolization in elongating spermatids. Failed spermiation at stages IX-X is indicated by asterisks (*). L, leptotene; Z, zygotene; P, pachytene; D, diplotene; RS, round spermatids; EL, elongating spermatids. Scale bar: 20 μm. (**E**) Scanning electron microscopy (SEM) reveals pronounced head abnormalities in mature SDKO sperm. Transmission electron microscopy (TEM) images of step 11–15 spermatids illustrate the abnormal nuclear condensation process in SDKO. The regions of interest are enlarged and highlighted with red rectangles, showing significantly less compact chromatin in mutant. Scale bar: 1 μm. (**F**) Western blot analysis of chromatin remodeling factors (core histones H1/H2A/H3/H4, transition proteins TNP1/TNP2, protamines PRM1/PRM2) in round spermatids, elongating spermatids, and epididymal sperm shows persistent histone retention and altered TNP2/PRM2 levels in SDKO versus controls. (**G**) Immunofluorescence detection of histone retention in SDKO mature sperm. Epididymal sperm smears from control and SDKO mice were stained for core histones (H2A, H3, H4). SDKO sperm nuclei show pronounced histone retention (red), where histones are effectively removed. Scale bar: 10 μm.

Previous single knockout studies showed that loss of either *Pum1* or *Pum2* alone impairs spermatogenesis but does not abolish fertility (*32*, *34*, *36*). To test whether PUM1 and PUM2 function redundantly in germ cells, we generated germ cell-specific double knockouts (DKOs) by deleting *Pum1* on a *Pum2*-null background at three developmental stages using *Vasa-Cre* (VDKO, embryonic germ cells) (*51*), *Stra8-Cre* (SDKO, spermatogonia) (*52*), and *Hspa2-Cre* (HDKO, spermatocytes) (*53*). Immunostaining confirmed efficient depletion of PUM1 in the appropriate cell types (Fig. 2, A and B).

All three conditional DKO strains exhibited markedly smaller testes, severe oligoasthenoteratozoospermia (OAT; reduced sperm count and motility, and abnormal morphology), and complete male sterility (Fig. 2C). The earlier the deletion, the stronger the reduction in sperm count and motility, with VDKO testes showing a ∼98% decline in sperm output. Thus, germ cell PUM1/2 are essential for normal sperm production and male fertility, with a knockout phenotype reminiscent of OAT observed in infertile men.

### Loss of PUM1/2 disrupts spermiogenesis and histone-protamine transition

Histological and ultrastructural analyses revealed two hallmark defects across all germline conditional double knockout (cDKOs): abnormal spermatid morphology and progressive germ cell loss. In SDKO (*Stra8-Cre;Pum1^f/f^;Pum2^−/−^*) testes, round spermatids appeared grossly normal, but elongating spermatids displayed severe nuclear deformation and vacuolization (Fig. 2, D and E and fig. S3, A and B). Epididymal spermatozoa showed dramatic head and tail abnormalities, with only ∼18% normal morphology compared to >90% in WT (fig. S3C).

Given the enrichment of chromatin regulators among PUM1/2 targets, we examined whether these defects reflected impaired chromatin remodeling. In WT testes, histones are efficiently replaced by transition proteins (TNPs) and protamines during spermiogenesis. In SDKO testes, core histones (H2A, H3, H4) were aberrantly retained in elongating spermatids and even mature spermatozoa, accompanied by elevated TNP2 and PRM2 levels (Fig. 2, F and G). This abnormal retention likely reflects defective histone removal and protamine balance, known causes of infertility. Consistent with the higher expression of PUM1 in meiotic germ cells and its disproportionate contribution to chromatin and spermiogenic defects, *Pum1* single knockout mice on a pure C57BL/6 background exhibited histone retention and sperm head abnormalities (fig. S3, D to G), whereas comparable defects were not observed in *Pum2* single knockouts, supporting a greater functional contribution of PUM1 during these stages.

### PUM1/2 are required for meiotic progression but dispensable for SSC maintenance

We next investigated the basis of germ cell loss in cDKOs. H&E staining, immunohistochemistry for the germ cell marker VASA, and TUNEL assays for apoptosis all revealed widespread depletion of germ cells in cDKO tubules, including not only postmeiotic spermatids but also a significant fraction of spermatocytes (fig. S4A).

To examine meiotic progression directly, we performed chromosome spreads and immunostained for SYCP3, a chromosome axis marker, together with γH2AX, a marker of meiotic double-strand breaks (DSBs). In WT spermatocytes, γH2AX foci are broadly distributed during leptotene and zygotene but are removed from autosomes as DSBs are repaired, remaining only on the sex body by pachytene. In contrast, a substantial subset of SDKO spermatocytes showed completed homolog pairing and chromosome condensation resembling pachytene yet retained γH2AX on autosomes (fig. S4, B and C). These spermatocytes, which displayed “pachytene-like” chromosome morphology but retained autosomal γH2AX, represented ∼14.1% of SDKO cells compared to ∼3.5% in WT. The persistence of γH2AX on autosomes indicates defective DSB repair and a delay in the zygotene-to-pachytene transition. Such aberrant spermatocytes are likely eliminated by apoptosis, accounting for the increased spermatocyte loss observed in SDKO testes. This meiotic defect was also evident in *Pum1* single knockouts on a pure C57B6 background (fig. S3G), consistent with the higher spermatocyte expression and dominant role of PUM1.

Importantly, comparable germ cell loss and spermatid morphological defects were observed across all three conditional knockout models (VDKO, SDKO, and HDKO), indicating that although the timing of deletion influences severity, loss of *PUM1/2* at multiple developmental stages converges on shared meiotic and postmeiotic defects. While meiotic defects were most readily quantified in SDKO testes due to efficient recovery of spermatocytes, VDKO and HDKO testes exhibited qualitatively similar reductions in spermatocyte populations and downstream spermiogenic failure.

By contrast, SSC populations were preserved in VDKO testes. PLZF and LIN28A staining confirmed the persistence of undifferentiated spermatogonia up to 14 months of age (fig. S4D). This finding indicates that PUM1/2 are dispensable for SSC maintenance, in striking contrast to *Drosophila* and *C. elegans* PUM homologs, which are required for germline stem cell survival (*29*–*31*). Thus, the conserved role of metazoan PUM proteins appears to lie in safeguarding germline continuity, but in mammals, this requirement has shifted from SSCs to meiotic and postmeiotic germ cells.

### PUM1/2 loss disrupts transcriptome integrity and reduces translation efficiency of chromatin regulators

To assess the molecular consequences of PUM1/2 deficiency, we profiled transcriptomes and translatomes of purified pachytene spermatocytes and round spermatids from WT and SDKO testes. RNA-seq revealed extensive dysregulation at both stages, with >2000 genes differentially expressed in each (fig. S5, A to C and table S3). Round spermatids exhibited more severe perturbations, likely reflecting cumulative effects of earlier meiotic defects. However, only ∼2% of differentially expressed genes carried PUM1/2 eCLIP peaks, indicating that most transcriptional changes are indirect. GO analysis of altered transcripts in round spermatids highlighted broad categories such as ion transport and vasculature development rather than spermatogenesis-specific pathways (fig. S5D), consistent with secondary consequences of impaired chromatin regulation.

**Fig. 3.**
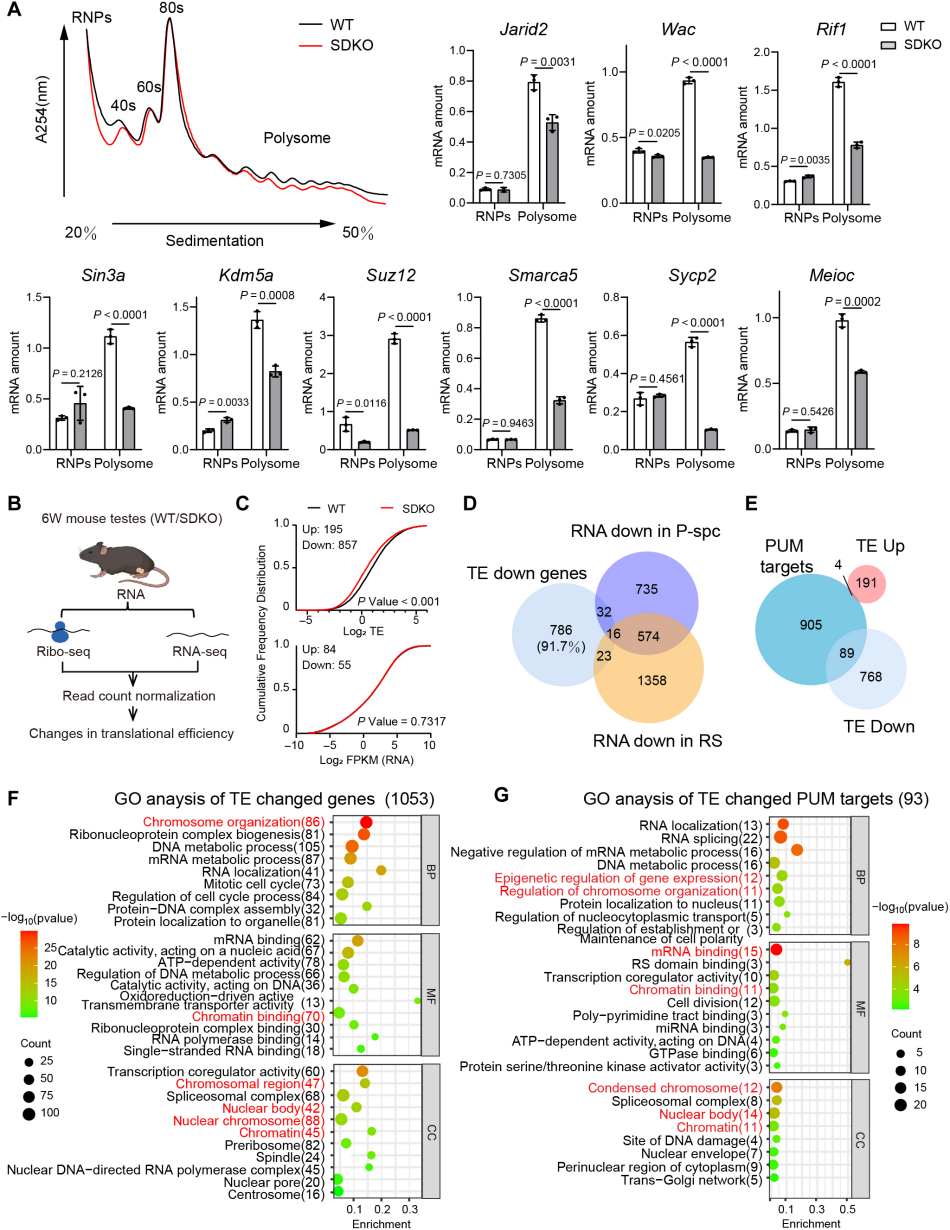
PUM1/2 deficiency broadly reduces translational efficiency and preferentially disrupts chromatin-regulatory mRNA translation. (**A**) Reduced polysome association of PUM1/2 target mRNAs in SDKO testes. Polysome profiling combined with RT-qPCR shows decreased loading of representative target mRNAs onto polysomes in SDKO versus control testes. Quantitative analysis is shown (mean ± SD; Student’s *t* test). (**B**) Schematic workflow of ribosome profiling (Ribo-seq) employed in this study. (**C**) Genome-wide shift toward reduced translation efficiency (TE) in SDKO testes. Top panel shows changes in ribosome occupancy measured by Ribo-seq; bottom panel shows changes in mRNA abundance measured by RNA-seq. “Up” and “Down” indicate significantly increased or decreased values (|log2 FC| > 1, FDR < 0.05). TE was calculated as the ratio of ribosome-protected fragment density to mRNA abundance. The distribution of TE log2 fold changes reveals a global shift toward reduced translation in SDKO testes (P < 0.001, Mann–Whitney test). (**D**) PUM1/2-mediated translational regulation is uncoupled from transcriptional changes in SDKO. Venn diagrams show minimal overlap between genes with decreased translational efficiency (TE-down) and those with reduced mRNA levels (RNA-seq down) in Pachytene spermatocytes (P-spc) or round spermatids (RS). (**E**) Almost all overlapping PUM targets with TE significantly changed (89 out of 93) have reduced translation efficiency in absence of *Pum1/2*. Venn diagram illustrating the overlap between genes with decreased TE and direct PUM1/2 eCLIP targets. (**F**) Chromatin-related pathways are enriched among translationally dysregulated genes. GO analysis of all genes with altered TE upon PUM1/2 loss shows significant enrichment for chromatin-associated biological processes. Enriched terms are categorized as Biological Process (BP), Molecular Function (MF), and Cellular Component (CC). (**G**) GO analysis of direct PUM1/2 targets with altered TE reveals enrichment in chromatin binding, epigenetic regulation and RNA regulation.

Given the limited effect of PUM1/2 loss on steady-state mRNA levels of direct PUM-bound targets as assessed by RNAseq (only ∼2% of differentially expressed genes carried PUM eCLIP peaks), we next examined translational control. To our surprise, loss of *Pum1* and *Pum2* in germ cells did not lead to de-repression of PUM target translation, based on the canonical model of PUMILIO protein (*42*). Instead, polysome profiling showed markedly reduced polysome association of multiple PUM1/2-bound chromatin regulators, including *Jarid2*, *Suz12*, *Sin3a*, *Smarca5* and *Kdm5a*-in SDKO testes despite stable mRNA levels (Fig. 3A).

Ribosome profiling revealed widespread alterations in translation efficiency, including a significant subset of direct PUM1/2 targets enriched for chromatin regulatory functions. Translation efficiency (TE) was significantly reduced for ∼900 genes, with 81.5% showing decreased TE in SDKO compared to WT (Fig. 3, B to D, tables S4 and S5). Only a minority of TE-affected genes were direct PUM1/2 targets (∼10%), suggesting that loss of PUM1/2 initiates widespread translational collapse through both direct and indirect mechanisms (Fig. 3E and table S6) and mode of PUM1/2 translational control in germ cells appears to be positive regulation rather than repression.

Although the majority of global TE changes in PUM1/2-deficient testes occur in transcripts not directly bound by PUM proteins, analysis of the subset of direct PUM targets with altered TE revealed strong functional enrichment for chromatin-associated regulators. Notably, ∼32% (30/89) of TE-reduced direct targets encode chromatin or chromosome regulatory factors (table S4). Consistent with this, GO cellular component analysis identified condensed chromosome, nuclear body, and chromatin as the top enriched categories (Fig. 3, F and G and table S7). Given the central regulatory roles of chromatin-associated factors, even selective translational disruption of this subset would be expected to propagate widespread secondary effects on the germ cell translatome.

**Fig. 4.**
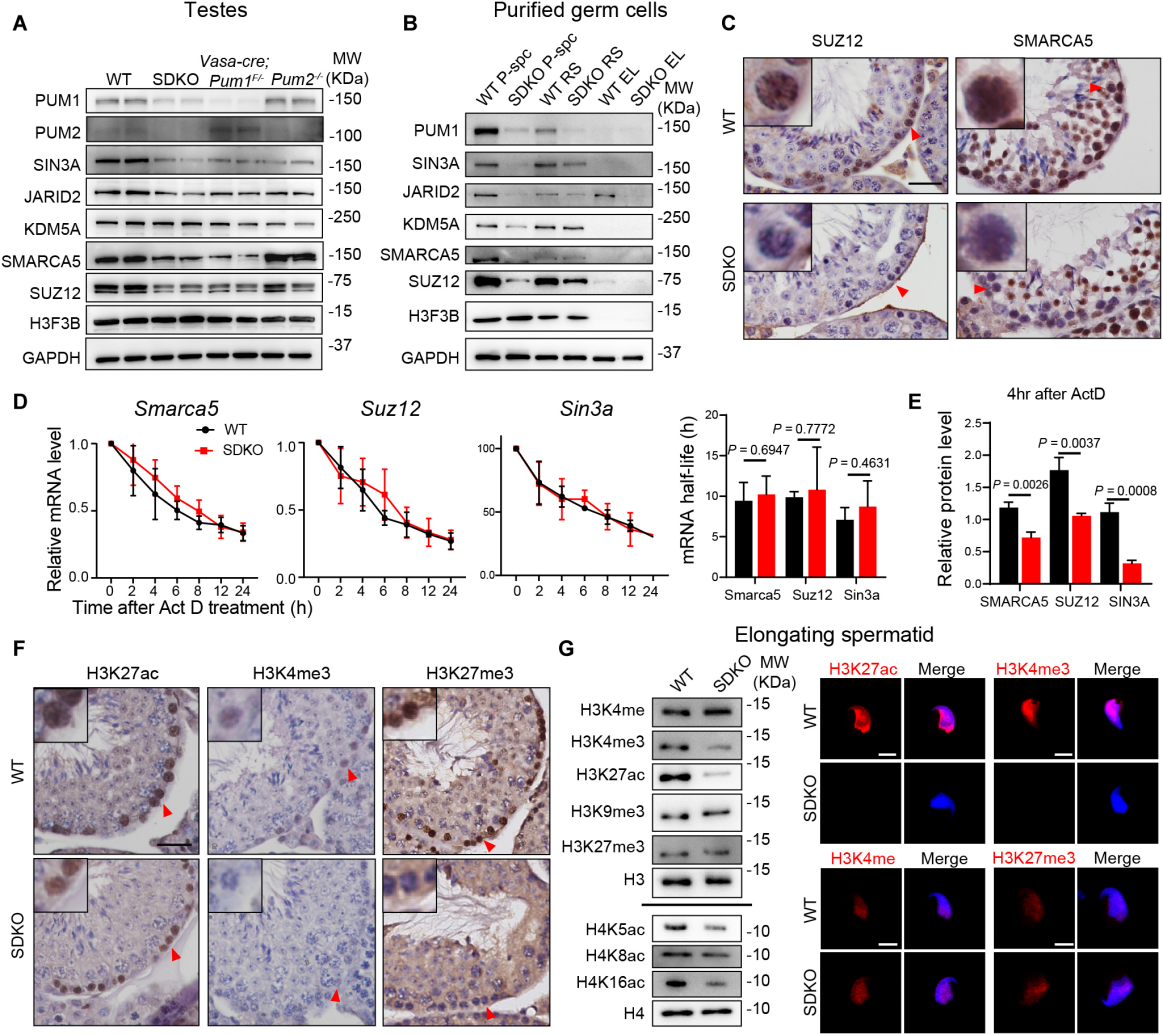
PUM1/2 loss reduces chromatin-regulator protein output without altering mRNA stability and disrupts stage-specific histone modifications during spermatogenesis. (**A** and **B**) Immunoblot analysis shows reduced protein levels of chromatin regulators in SDKO samples compared to wild-type controls, including whole-testis lysates (A) and purified germ cells (P-spc, RS and EL) (**B**). Germ cell-specific *Pum1* knockout (*Vasa-cre;Pum1^f/−^*) and *Pum2^−/−^* testes are included for comparison. GAPDH serves as a loading control. (**C**) Immunohistochemical detection of SUZ12 and SMARCA5 in testes. Marked reduction of SUZ12 and SMARCA5 signals is observed in spermatocytes (indicated by black and red arrowheads, respectively) of SDKO testes compared with WT controls. Scale bar: 20 μm. (**D**) mRNA decay kinetics, mRNA half-life of *Smarca5*, *Sin3a*, and *Suz12* in cultured seminiferous tubules isolated from WT and SDKO testes following transcriptional inhibition with actinomycin D are shown respectively. The mRNA levels were measured by RT–qPCR at the indicated time points (0–24 h), normalized to time zero, and plotted as relative abundance. Decay curves were fitted using exponential regression to estimate mRNA half-lives from three biological replicates. No significant difference in mRNA half-life was detected between WT and SDKO samples for any of the three transcripts (*P* > 0.05). 18S rRNA served as the normalization control. (**E**) The quantification of PUM-targeted chromatin-regulator protein levels (see fig. S5F) show relative band intensities quantified with ImageJ. Despite comparable mRNA decay kinetics, protein abundance of these targets was reduced in SDKO samples, consistent with impaired translational output rather than altered mRNA stability. (**F**) Immunohistochemical detection of H3K27ac, H3K4me3, and H3K27me3 in spermatocytes of WT and SDKO testes. Arrowheads indicate the cells shown in the enlarged insets. Scale bar: 20 μm. (**G**) Western blot and immunofluorescence analysis of key histone modifications in elongating spermatids (RS). Scale bar for immunofluorescence images: 5 μm.

Protein-level analyses substantiated these translational defects. Western blotting and immunohistochemistry revealed significant reductions in SIN3A, SUZ12, SMARCA5, and JARID2, particularly in spermatocytes where PUM1/2 are most highly expressed (Fig. 4, A to C). The reduced protein output of PUM targets could, in principle, result from altered mRNA stability (*42*). To determine if mRNA stability might contribute to reduction of target protein output in absence of *Pum1/2*, we quantified mRNA decay kinetics in cultured seminiferous tubules from WT and SDKO testes at various time points following Actomycin D treatment. Exponential regression analysis of decay curves from three biological replicates revealed no significant difference in half-life for *Suz12*, *Sin3a*, or *Sycp2* (Fig. 4D), yet protein output from the three target mRNAs is consistently reduced (Fig. 4D and fig. S6A). This finding argues against altered mRNA decay as the primary mechanism.

We further explored the impact of loss of those key chromatin regulators in the mutant testes by examining the epigenetic modification of histone proteins using specific antibodies. These loss or reduction of those chromatin regulators were accompanied by global epigenetic abnormalities: reduced H3K27ac, H3K4me3, and H4 acetylation in the mutant testis (Fig. 4F) and in elongating spermatids (Fig. 4G), likely contributing to histone retention and disrupted histone-protamine transition. Together, these findings demonstrate that PUM1/2 primarily act to promote translation of a subset of key chromatin regulators, and that their absence leads to epigenetic disruption, defective meiosis, and impaired sperm maturation.

### PUM1/2 associate with DAZL and PABPC1 and converge on shared chromatin-regulator mRNAs in testis

**Fig. 5.**
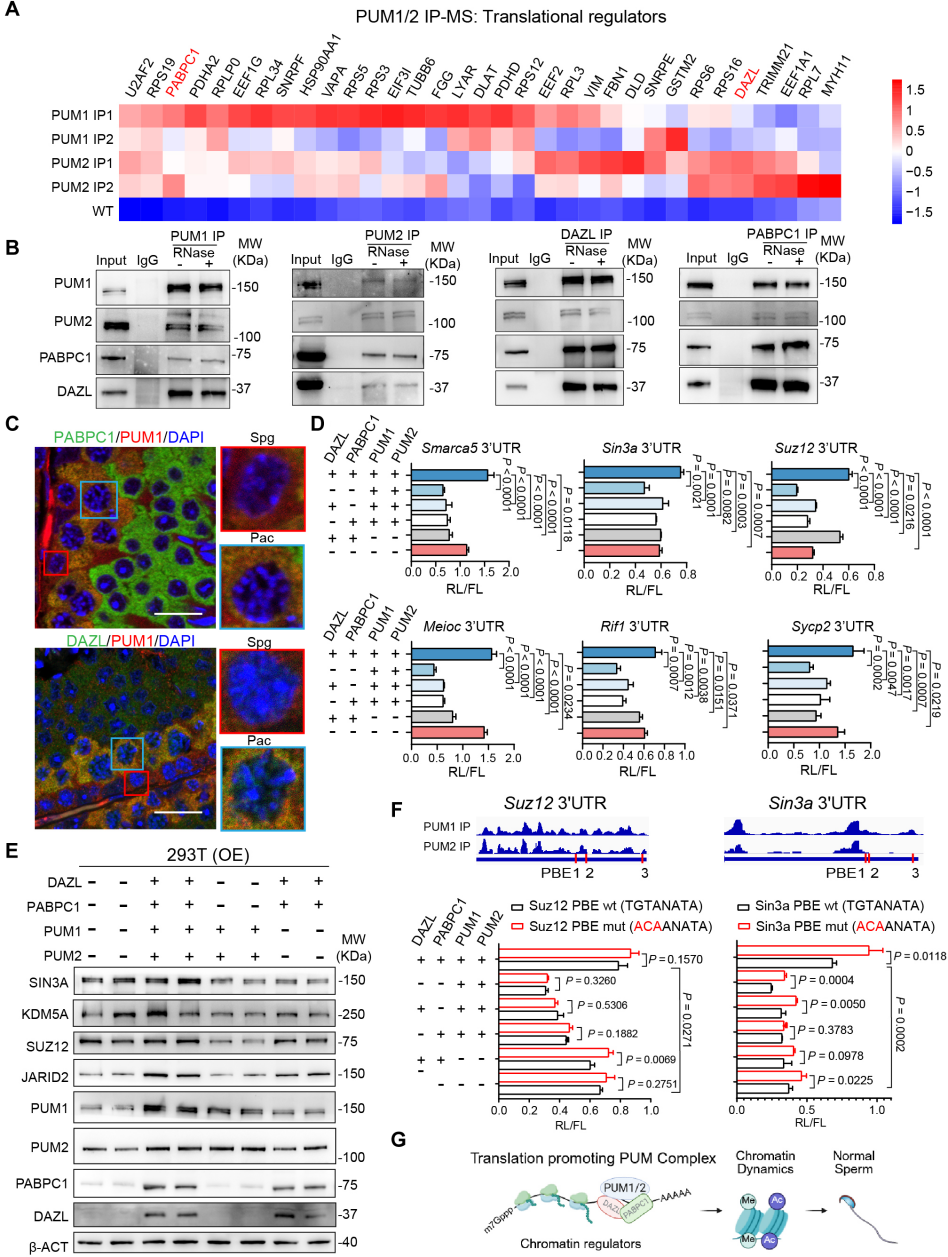
PUM1 and PUM2 associate with DAZL and PABPC1 and converge on shared chromatin-regulator mRNAs in testis. (**A**) IP–MS from adult testes of *Pum1-HA* and *Pum2-HA* knock-in mice identifies interactors enriched for RNA binding and translational regulation, including DAZL and PABPC1. See also fig. S7, A and B and table S8. (**B**) Co-immunoprecipitation (Co-IP) analysis confirms association of endogenous PUM1 and PUM2 with DAZL and PABPC1 in adult testes. RNase A treatment indicates these interactions are largely RNA-independent. (**C**) Immunofluorescence of adult testes reveals cytoplasmic co-localization of PUM1/2 with DAZL and PABPC1 in pachytene spermatocytes (Pac), but minimal signal in spermatogonia (Spg). DAPI, blue. Scale bar, 20 μm. (**D**) Dual-luciferase assays using selected PUM target 3′UTRs show that PUM1 or PUM2 alone modestly repress translation, whereas co-expression with DAZL and PABPC1 increases reporter expression. RL/FL: Renilla/firefly luciferase ratio. Data are mean ± SD. (**E**) Western blot analysis of HEK293T cells co-expressing PUM1, PUM2, DAZL, and PABPC1 shows increased levels of selected chromatin regulators. β-actin serves as a loading control. (**F**) Top, IGV tracks showing PUM1 and PUM2 eCLIP peak on the 3′UTRs of *Suz12* and *Sin3a*, with annotated canonical PBE positions. Bottom, dual-luciferase assays using wild-type or PBE-mutant 3′UTRs. Mutation of the annotated canonical PBE partially reduces basal repression by PUM1/2 but does not abolish increased reporter expression in the presence of DAZL and PABPC1, indicating that reporter responsiveness is not fully explained by a single canonical PBE. See also fig. S7K. (**G**) Model illustrating a testis-specific translational regulatory network in which PUM1/2 bind chromatin-regulator mRNAs in vivo, while DAZL and PABPC1 associate with selected shared targets.

To investigate how PUM1/2 promote rather than repress translation, we defined the PUM1/2 interactome by IP-LC/MS using HA-tagged knock-in testes. Both PUM1 and PUM2 associated with factors involved in RNA regulation and translation, including DAZL and PABPC1 (*24*) (Fig. 5A, fig. S7, A and B, and table S8). Co-IP confirmed that PUM1/2 physically interact with DAZL and PABPC1 in an RNA-independent manner (Fig. 5B), and immunofluorescence revealed cytoplasmic colocalization in spermatocytes, especially in pachytene spermatocytes, consistent with a functional interaction in meiotic germ cells (Fig. 5C). Importantly, DAZL and PABPC1 expression levels and their interactions were not disrupted in SDKO testes (fig. S7, C and D). Moreover, the distribution profiles of DAZL and PABPC1 across polysome fractions showed no significant changes upon PUM depletion compared to those in WT testes (fig. S7E), indicating that PUM1/2 loss disrupts function rather than abundance of these co-factors.

Next we investigate the direct regulation of PUM1/PUM2 complex via 3’UTR of selected chromatin targets (*Smarca5*, *Sin3a*, *SUZ12*, *Meioc*, *Rif1*, *Sycp2*) (Fig. 5D) and non-PUM targets (*Mei4*, *Sycp3*) and PUM target with no TE change (*Sycp1*) (fig. S7F) in 293 T cells. We compare the Renilla Luciferase (RL) normalized against internal control Firefly Luciferase (FL) to evaluate the translation output after adding individual components of PUM1/2-DAZL-PABPC1 components and all four components. PUM1 and PUM2 alone repressed translation of 3′UTRs from chromatin regulator targets, but co-expression with DAZL and PABPC1 converted repression into strong activation, with up to a twofold increase in luciferase activity (Fig. 5D). Even comparing with expression of DAZL and PABPC1 along, additional expression of PUM1, PUM2 will significantly increase the translational output of the reporters for all six PUM1/2 target genes (*Smarca5*, *Sin3a*, *SUZ12*, *Meioc*, *Rif1*, *Sycp2*) (Fig. 5D) but not for non-PUM targets or PUM target with no TE change (fig. S7F). Consistent with cooperative paralog function, robust activation required co-expression of both PUM1 and PUM2 together with DAZL and PABPC1, whereas either paralog alone showed minimal activation (fig. S7G). Western blots confirmed that co-expression of all four factors enhanced protein levels of SUZ12, SIN3A, KDM5A, and JARID2 (Fig. 5E) but did not alter mRNA decay kinetics (fig. S6, B to D), indicating that increased protein output primarily reflects enhanced translation rather than altered RNA stability. These data support functional interplay among PUM1/2, DAZL, and PABPC1, although the precise cis-regulatory logic underlying this effect remained unclear.

Because PBE was the most enriched motif in our PUM1/2 eCLIP datasets, we next asked whether annotated canonical PBEs contribute to reporter responsiveness of representative targets. We focused on *Suz12* and *Sin3a*, two chromatin-regulator mRNAs directly bound by PUM1/2 in vivo. IGV analysis showed that annotated canonical PBEs are present in the 3′UTRs of both genes but are not centered within the strongest PUM1/2 eCLIP peaks (Fig. 5F, top). When we mutated the annotated PBE in the corresponding reporter constructs, basal repression by PUM1/2 was partially reduced; however, increased reporter expression in the presence of DAZL and PABPC1 was retained (Fig. 5F, bottom). These findings suggest that canonical PRE/PBE sequences contribute to basal repression but are not strictly required for translational activation in this reporter context.

Consistent with a modulatory rather than recruitment role for PUM1/2, RIP-qPCR revealed that DAZL association with target mRNAs was largely preserved in SDKO testes, whereas PABPC1 enrichment was selectively reduced for a subset of transcripts (fig. S7H). Among genes with reduced translation efficiency (TE) in SDKO testes, ∼10% are direct PUM1/2 eCLIP targets, whereas an additional ∼60% are bound by DAZL or PABPC1 based on published CLIP datasets (*24*, *54*) (fig. S7I). Together, these data support a model in which PUM1/2 act to potentiate translational output from a subset of DAZL-bound transcripts, rather than serving as primary determinants of DAZL substrate binding.

This interpretation is consistent with our broader eCLIP analysis, which showed that many PUM1/2 peaks in testis are not centered on canonical PRE/PBE sequences. We therefore infer that PUM1/2 target recognition and regulatory output in germ cells are more flexible than a simple PRE/PBE-only model and likely depend on broader 3′UTR context and/or cooperative RNP assembly. To directly test whether Suz12 and Sin3a are associated with DAZL and PABPC1 in the relevant tissue context, we performed RIP-qPCR in adult mouse testes and found that both mRNAs were recovered in DAZL and PABPC1 immunoprecipitates (fig. S7J). Together, these data support a model in which PUM1/2, DAZL, and PABPC1 converge on a shared set of target mRNAs in testis, while the precise cis-elements mediating this cooperation remain to be fully defined.

Together, these results support the model illustrated in Fig. 5G, in which PUM1/2 bind chromatin-regulator mRNAs in vivo, while DAZL and PABPC1 associate with selected shared targets in testis.

## DISCUSSION

Our study uncovers an essential and unexpected role for mammalian PUM1 and PUM2 in promoting translation of chromatin regulators to ensure successful spermatogenesis. Traditionally, PUM proteins are viewed as canonical translational repressors and mRNA decay factors (*42*, *43*). However, we show that in the mammalian male germline, PUM1/2 act as translational activators, partnering with DAZL and PABPC1 to promote efficient translation of chromatin regulators such as SUZ12, SIN3A, JARID2, and SMARCA5, thereby safeguarding the production of epigenetic regulators critical for meiotic progression and chromatin remodeling. Loss of PUM1/2 compromises translation efficiency of these targets, leading to disrupted meiotic progression, impaired histone–protamine transition, widespread transcriptome perturbations, and infertility. These findings expand the functional repertoire of PUM proteins and reveal a new translational layer of control in epigenetic regulation during spermatogenesis.

### PUM proteins as context-dependent translational activators in the germline

The prevailing model positions PUM1/2 as post-transcriptional repressors, based on structural studies of the PUM domain and transcriptome-wide mapping of PUM-mRNA interactions (*28*, *42*–*44*). Yet mounting evidence suggests that PUMs can also activate translation in specific contexts (*45*, *55*). In hematopoietic progenitors, PUM1/2 enhance FOXP1 expression (*45*), and in neurons, PUM1 dosage sensitivity affects translational regulation of synaptic mRNAs via its context-dependent interactions with other proteins (*56*). Our findings extend this functional versatility to germ cells: PUM1/2 promote translation of chromatin regulators such as *Suz12, Sin3a, Smarca5* and *Jarid2* with little impact on their mRNA stability. Mechanistically, this activity depends on their partnership with DAZL and PABPC1, two well-established translational activators in germ cells (*24*, *25*, *57*, *58*). We propose that PUM1/2 binding facilitates recruitment or stabilization of the DAZL/PABPC1 translational machinery, thereby shifting PUM function from repression to activation. This context-dependent “functional switch” underscores the plasticity of RNA-binding proteins and highlights the unique translational environment of germ cells (*21*, *42*, *59*). In this context, our findings provide a functional framework for earlier observations of PUM–DAZ family protein interactions in human germ cells (*60*), establishing their cooperative role in promoting translation in vivo.

At the same time, our data support a model in which PUM1/2 operate within a specialized translational subnetwork rather than globally controlling DAZL activity. DAZL–PABPC1 interaction, DAZL and PABPC1 protein levels, and their polysome distribution are largely preserved in SDKO testes (fig. S7, C to E), indicating that PUM1/2 are not required for DAZL expression or its general translational functions. Instead, loss of PUM1/2 selectively reduced translation efficiency and PABPC1 association for a subset of target transcripts, particularly chromatin regulators. Together, these results support the model illustrated in Fig. 5G, in which PUM1/2 function not as global determinants of DAZL binding, but as facilitators of maximal translational activation within a specialized subset of DAZL- and PABPC1-associated germ cell transcripts, including chromatin regulators.

A notable outcome of our mechanistic analysis is that canonical PRE sequences do not fully explain the in vivo PUM1/2 binding landscape in testis. Although PRE/PBE was the most enriched motif in our eCLIP datasets, many PUM1/2 peaks were not centered on canonical PRE/PBE sites, including peaks on representative chromatin-regulator targets such as *Suz12* and *Sin3a*. Consistent with this, mutation of annotated canonical PBEs in selected 3′UTR reporters partially relieved basal repression by PUM1/2 but did not abolish increased reporter expression in the presence of DAZL and PABPC1. These findings indicate that canonical PRE/PBE motifs contribute to regulation but do not fully account for the translational behavior of all targets in germ cells. Instead, PUM1/2 target recognition and regulatory output in testis likely depend on broader 3′UTR context and/or cooperative RNP assembly. Supporting this interpretation, *Suz12* and *Sin3a* are also recovered in DAZL and PABPC1 immunoprecipitates from testis, indicating convergence of these factors on shared target mRNAs in the relevant tissue context.

### Translational control of chromatin regulators during meiosis and spermiogenesis

A striking feature of PUM1/2 targets is their enrichment for chromatin regulators, including histone modifiers, nucleosome remodelers, and recombination factors. This finding directly links translational control to two critical germline transitions: meiotic progression and spermatid chromatin remodeling.

Although PUM1/2-deficient spermatocytes exhibit delayed progression through meiosis and persistence of autosomal γH2AX, these defects are partial and do not resemble a classical meiotic checkpoint arrest. Rather than directly regulating checkpoint signaling pathways, our data support a model in which PUM1/2 promote efficient meiotic progression indirectly, by ensuring adequate translational output of chromatin regulators required for proper chromatin organization and DNA repair dynamics. Disruption of this translational program likely sensitizes a subset of spermatocytes to elimination, contributing to the observed germ cell loss.

At the spermatid stage, loss of PUM1/2 caused defective histone eviction, reduced H3K27ac and H3K4me3, and abnormal histone retention in mature sperm. These defects parallel phenotypes seen in *Dot1l* mutant mice, where transcriptional regulation of histone eviction factors and histone–protamine transition are disrupted (*12*, *15*). Together with recent work showing roles for CTCF-mediated 3D chromatin organization (*11*, *16*), our findings highlight a tightly interwoven network of transcriptional, epigenetic, and translational mechanisms that coordinate chromatin remodeling during spermatogenesis. We propose that PUM1/2 provide a translational “fail-safe” mechanism to ensure chromatin regulators are supplied during transcriptionally silent stages.

Notably, reanalysis of published PUM1/2 CLIP datasets from somatic cell lines also revealed chromatin regulators as a significantly enriched target class. While additional validation is required, this suggests that translational control of chromatin factors may represent a general and conserved role of PUM proteins beyond the germline.

### Developmental deployment and asymmetric contribution of PUM1 and PUM2

Our study also clarifies both the developmental timing and the relative contribution of PUM1 and PUM2 during spermatogenesis. Our results suggest that the regulatory mode of PUM1/2 is deployed primarily during meiosis and early spermiogenesis rather than during spermatogonial stem cell maintenance. PUM1 expression peaks in pachytene spermatocytes, coinciding with robust cytoplasmic colocalization and physical interaction with DAZL and PABPC1, whereas spermatogonial stem cells remain intact following combined *Pum1/2* loss. Moreover, translational defects, chromatin dysregulation, and germ cell loss emerge at meiotic and postmeiotic stages, consistent with a stage-restricted requirement for PUM-dependent translational activation.

These findings are particularly notable in light of the deeply conserved roles of Pumilio proteins in invertebrate germline stem cell maintenance and fate specification (*19*, *29*–*31*, *41*). Our germ cell-specific double knockout analysis directly tests whether these stem cell-associated functions are conserved in mammals. Surprisingly, combined loss of *Pum1* and *Pum2* did not compromise long-term maintenance of spermatogonial stem cells, even when deletion occurred in embryonic germ cells. Instead, the dominant requirement for PUM1/2 emerged during meiotic and postmeiotic stages, where they safeguard chromatin remodeling and sperm differentiation. These findings indicate that, although Pumilio proteins retain essential roles in germline continuity across metazoans, their developmental deployment has diverged in mammals, shifting from stem cell maintenance toward regulation of meiotic progression and spermiogenesis.

Our study also clarifies the relationship between PUM1 and PUM2. While previous mammalian studies typically examined one paralog at a time (*32*, *36*–*39*), we demonstrate that both paralogs contribute to fertility but with unequal weight. PUM1 exerts a dominant role: its loss alone produces defects in meiotic progression, histone retention, and sperm morphology, whereas PUM2 loss yields milder effects. This asymmetric redundancy likely reflects its higher abundance in spermatocytes. Analogous redundancy has been described in *C. elegans*, where the PUM homologs FBF-1 and FBF-2 act together to maintain germline stem cells (*29*, *61*). The conservation of paralogous PUF proteins across metazoans suggests selective pressure to maintain dosage-sensitive translational regulation. In mammals, this system appears to have evolved toward PUM1 dominance.

### Broader implications

Our findings add to a growing recognition that translational control is a central regulatory layer in spermatogenesis (*20*, *22*–*24*, *26*, *62*, *63*). Similar to MIWI/piRNA and FXR1, which promote translation of stored mRNAs in spermatids (*22*, *23*), PUM1/2 safeguard the translational output of key chromatin regulators at earlier meiotic stages. The convergence of multiple RBPs on translational activation rather than repression highlights a unique germline adaptation in which canonical repressors are repurposed as activators to meet the challenges of meiosis and genome reorganization (*3*, *64*, *65*).

Notably, the germ cell-specific *Pum1/2* knockout phenotype strongly resembles human OAT, characterized by reduced sperm count, weak motility, and abnormal morphology. Given that OAT is a major cause of idiopathic male infertility, our findings raise the possibility that dysregulation of PUM-mediated translational control could represent an underappreciated mechanism contributing to defective spermatogenesis in humans. More broadly, because aberrant PUM activity has been implicated in infertility, cancer, and neurodevelopmental disorders (*37*, *49*, *55*), our work underscores the biomedical importance of understanding how PUM proteins toggle between translational repression and activation.

In summary, we establish that mammalian PUM1 and PUM2 are indispensable for spermatogenesis by promoting translation of chromatin regulators in the context of a germ-cell translational network that includes DAZL and PABPC1. This mechanism secures meiotic progression, chromatin remodeling, and sperm maturation. Our results redefine PUM1/2 as context-dependent translational activators in the germline, reveal an asymmetric collaboration between PUM1 and PUM2, and position PUM proteins as central integrators linking RNA regulation to chromatin dynamics.

#### Declaration of generative AI and AI-assisted technology

During the preparation of this work the authors used ChatGpt 4.1 in order to increase manuscript’s readability. After using this tool/service, the authors reviewed and edited the content as needed and take full responsibility for the content of the published article.

## MATERIALS AND METHODS

### Mice and genotyping

*Pum1-HA* and *Pum2-HA* knock-in mice were generated by CRISPR/Cas9-mediated insertion of an HA epitope tag at the C terminus. *Pum1* and *Pum2* knockout mice were generated as described before (*33*). Germ cell-specific conditional knockouts were produced by crossing floxed *Pum1* alleles with *Pum2*-null mice and germline stage-specific Cre drivers: Vasa-Cre (embryonic germ cells), Stra8-Cre (premeiotic spermatogonia), and Hspa2-Cre (spermatocytes), resulting in Vasa-Cre (VDKO mutant), Stra8-Cre (SDKO mutant), or Hspa2-Cre (HDKO mutant) in the *Pum1^f/f^;Pum2^−/−^* or *Pum1^f/−^;Pum2^−/−^* genetic background. Genotyping was performed by PCR with primers flanking the modified loci. All mice were maintained on C57BL/6 or mixed backgrounds as indicated. Animal experiments were approved by the Institutional Animal Care and Use Committee (IACUC) of Nanjing Medical University (No. IACUC-2406117). Mice were housed under a 12-h light/12-h dark cycle with free access to food and water.

### Histology, immunohistochemistry (IHC), and immunofluorescence (IF)

Immunoblot analysis of testis lysates from 2-month-old *Pum1-HA* and *Pum2-HA* knock-in mice to evaluate the relative protein abundance of PUM1 and PUM2. Relative protein levels were quantified by densitometric analysis of HA-tag signals using ImageJ

Testes were fixed in Hartman’s fixative or 4% paraformaldehyde, embedded in paraffin, sectioned (5 μm), and stained with hematoxylin and eosin (H&E). For IHC/IF, antigen retrieval was performed in citrate buffer (pH 6.0), followed by blocking in 5% bovine serum albumin. Primary antibodies included anti-PUM1, anti-PUM2, anti-VASA, anti-PLZF, anti-LIN28A, anti-SYCP3, anti-γH2AX, anti-SUZ12, anti-SMARCA5, anti-H2A, anti-H3, anti-H4, anti-H3K4me, anti-H3K4me3, anti-H3K27ac, anti-H3K27me3, anti-DAZL, anti-PABPC1 and anti-HA-tag. Secondary antibodies conjugated to Alexa Fluor dyes were used for IF; biotinylated secondaries and 3,3′-diaminobenzidine (DAB) were used for IHC. Nuclei were counterstained with DAPI. Images were acquired on Zeiss confocal or Leica DM6000 microscopes.

### Transmission electron microscopy (TEM)

Testes were fixed in 2.5% glutaraldehyde, postfixed in 1% osmium tetroxide, dehydrated through ethanol, and embedded in Epon resin. Ultrathin sections (70 nm) were stained with uranyl acetate and lead citrate and imaged on a JEOL JEM-1010 electron microscope.

### Scanning electron microscopy (SEM)

For SEM analysis, sperm-containing supernatant was centrifuged (300g, 5 min) to pellet cells. The pellet was resuspended in electron microscope fixative, fixed (RT, 2 h), then stored at 4°C. Samples were post-fixed in 1% OsO4 (0.1 M PBS, RT, 1-2 h), washed thrice with PBS (15 min each), and gradient dehydrated in ethanol. After isoamyl acetate treatment, samples were critical point dried, mounted on metal stubs with carbon tabs and imaged on a JEOL JSM-7900FLV-SEM electron microscope.

### Epididymal sperm analysis

The cauda epididymides were dissected and minced into ∼2 mm fragments in HTF medium to facilitate sperm release. After incubation for 5 min at 37°C, mature sperm from the supernatant were collected and analyzed for count, motility, and morphology. Sperm smears were stained with eosin, and morphological abnormalities were quantified from >200 sperm per sample by microscopy.

### TUNEL assay

Apoptotic germ cells were detected using the In Situ Cell Death Detection Kit (Vazyme) according to manufacturer’s instructions, followed by counterstaining with DAPI. Positive cells were quantified per seminiferous-tubule cross-section.

### Chromosome spreads

Spermatocyte spreads were performed as previously described (*24*). Cells were fixed in 1% paraformaldehyde containing 0.15% Triton X-100 and 0.5 M sodium tetraborate, air-dried, and immunostained with anti-SYCP3 and anti-γH2AX. Meiotic substages were classified based on SYCP3 morphology and γH2AX distribution. At least 200 spermatocytes per genotype were analyzed.

### Western blotting

Testes or purified germ cells were lysed in RIPA buffer supplemented with protease inhibitors. Proteins were resolved by SDS-PAGE, transferred to PVDF membranes, and probed with indicated antibodies. The primary antibodies were listed in the table S1. Chemiluminescent signals were detected using ECL (Thermo). Band intensities were quantified with ImageJ.

### Germ cell purification

Spermatocytes and round spermatids were isolated from adult mouse testes via sequential digestion with collagenase I and trypsin. The resulting single-cell suspension was separated by STA-PUT velocity sedimentation in a BSA gradient (*66*). Fractions were collected and examined under phase-contrast microscopy and fluorescent microscopy on DAPI-stained cells; pachytene spermatocytes and round spermatids were identified based on characteristic nuclear size and morphology (Pachytene spermatocytes are anywhere from 12–18 μm in diameter and consist of a thin rim of cytoplasm surrounding a large nucleus; Round spermatids are around 10 μm in diameter and have a round nucleus; Elongating spermatids have a sickle-shaped nucleus). Only fractions with >90% purity (assessed by counting >500 cells per fraction) were pooled for subsequent RNA-seq analysis.

### RNA isolation, RNA-seq and data analysis

Total RNA was extracted with TRIzol (Invitrogen), followed by DNase I treatment. RNA integrity was confirmed on an Agilent Bioanalyzer. Libraries were prepared using TruSeq stranded mRNA kit (Illumina) and sequenced on an Illumina HiSeq 2500. Reads were aligned to mm10 genome using STAR, and differential expression was analyzed with DESeq2. Gene Ontology (GO) enrichment was performed with Metascape.

### eCLIP

eCLIP was performed as described (*67*, *68*). Briefly, testes were UV-crosslinked (254 nm, 400 mJ/cm^2^), lysed, and immunoprecipitated with anti-PUM1 or anti-PUM2 antibodies. After stringent washes, RNA was ligated to adapters, reverse-transcribed, and amplified. Single-end sequencing was performed on an Illumina HiSeq 2500. Sequencing libraries were generated and analyzed using CLIPper and HOMER for peak calling and motif enrichment.

### Polysome profiling

Testes were homogenized in polysome buffer (20 mM Tris-HCl pH 7.4, 100 mM KCl, 5 mM MgCl_2_, 100 μg/ml cycloheximide, 1 mM DTT, 20 U/ml RNase inhibitor, and 1× protease-inhibitor cocktail). Lysates were layered onto 20–50% sucrose gradients and centrifuged at 38,000 rpm for 2 h. Gradients were fractionated with continuous A260 monitoring on BIOCOMP gradient machine (BioComp, S00501). RNA from fractions was extracted for qRT-PCR.

### Ribosome profiling (ribo-seq)

Ribo-seq was performed using TruSeq Ribo Profile kit (Illumina). Ribosome-protected fragments (28–32 nt) were isolated from RNase I-digested testis lysates, size-selected, and sequenced. Reads were mapped to coding sequences using Bowtie2, and translation efficiency was calculated as ribosome-footprint density normalized to mRNA abundance. All data analysis and processing were applied by Gene Denovo Biotechnology Co. (Guangzhou, China).

### RNA decay assay with cultured seminiferous tubules and HEK293T cells

In seminiferous tubules: Seminiferous tubules from adult WT and SDKO mice were prepared as described previously (*69*, *70*). Briefly, freshly dissected testes were treated with collagenase to isolate tubules, which were then cut into small segments and incubated in StemPro-34 SFM medium supplemented with corresponding supplements, 1% KnockOut Serum Replacement, 0.1 mM non-essential amino acids, and 1 mM sodium pyruvate for 1 h. Subsequently, tubules were treated with 20 μg/ml Actinomycin D for 24 h, with samples harvested at seven time points (0, 2, 4, 6, 8,12, and 24 hrs.) after the treatment.

In cell lines: HEK293T cells were transfected with empty vectors or vectors encoding PUM1/PUM2/DAZL/PABPC1. At 36 h post-transfection, Actinomycin D was added to a final concentration of 5 μg/ml.

Total RNA was extracted at each time point, reverse-transcribed, and analyzed by quantitative PCR using 18S rRNA as the internal control. mRNA levels were normalized to time 0 for each gene and plotted as relative abundance over time. Apparent mRNA half-life was calculated as the time required for transcript levels to decrease to 50% of the initial value based on decay curves. Three independent biological replicates were analyzed per genotype.

### Immunoprecipitation and mass spectrometry (IP-MS)

Testes from *Pum1-HA* and *Pum2-HA* knock-in mice were lysed in IP buffer (20 mM Tris-HCl pH 7.5, 150 mM NaCl, 0.5% NP-40). HA-tagged proteins were immunoprecipitated using anti-HA affinity beads (Sigma), eluted, and subjected to LC-MS/MS analysis, which was carried out by Shanghai APTBIO Co., Ltd. (Shanghai, China). Peptides were separated on an Easy-nLC 1000 UHPLC system (Thermo Fisher Scientific) and analyzed on a Q Exactive mass spectrometer (Thermo Fisher Scientific). The resulting mass spectrometry data were processed with MaxQuant and filtered against negative control samples.

### Dual-luciferase reporter assays

Luciferase reporters containing 3′UTRs of chromatin-regulator targets were cloned downstream of the firefly-luciferase gene. HEK293T cells were co-transfected with reporters and expression constructs for PUM1/2, DAZL, and PABPC1. Renilla luciferase activity was normalized to firefly luciferase. Experiments were performed in triplicate. Luminescence was measured on a BioTek Synergy 2 microplate reader. Specifically, HEK293T cells in 24-well plates were transfected with 150 ng of psi-CHECK-2 construct carrying targets 3’ UTR plus 450 ng of overexpression plasmid or control pCMV vector using ExFect Transfection Reagent (Vazyme). After 36–48 hr., the cells were collected to measure Firefly luciferase (FL) expression and Renilla luciferase (RL) expression using the Dual Luciferase Reporter Assay Kit (Vazyme). Renilla Luciferase was normalized to Firefly control. The results were analyzed by BioTek Synergy 2 microplate reader. All reporter assays were performed in three replicates.

### Quantification and statistical analysis

Statistical analysis was performed by GraphPad Prism 8.0 (GraphPad Software). All quantitative data are presented as mean ± SD. Statistical significance was determined by two-tailed Student’s *t* test (two groups) or one-way ANOVA with Tukey’s post hoc test (multiple groups). *P* < 0.05 was considered significant.
