# Supplementary material for: PUM1 and PUM2 promote translation of chromatin regulators to ensure mammalian spermatogenesis
Source: Sci Adv. 2026 Jul 17;12(29):eaed5708. doi: 10.1126/sciadv.aed5708 (PMC13378543; doi:10.1126/sciadv.aed5708)
Supplement: Supplementary file 1 — Figs. S1 to S7 Legends for tables S1 to S10 [file sciadv.aed5708_sm.pdf]

Science Advances
AAAS

Supplementary Materials for

PUM1 and PUM2 promote translation of chromatin regulators to ensure mammalian spermatogenesis

Min Zang *et al.*

Corresponding author: Bing Yao, yaobing@nju.edu.cn; Mingxi Liu, mingxi.liu@njmu.edu.cn; Kaibo Lin, linkb@126.com; Eugene Yujun Xu, yxu@uchicago.edu

Sci. Adv. **12**, eaed5708 (2026)
DOI: 10.1126/sciadv.aed5708

The PDF file includes:

Figs. S1 to S7
Legends for tables S1 to S10

Other Supplementary Material for this manuscript includes the following:

Tables S1 to S10

Zang et al. "PUM1 and PUM2 promote translation of chromatin regulators to ensure mammalian spermatogenesis"

Supplemental Materials

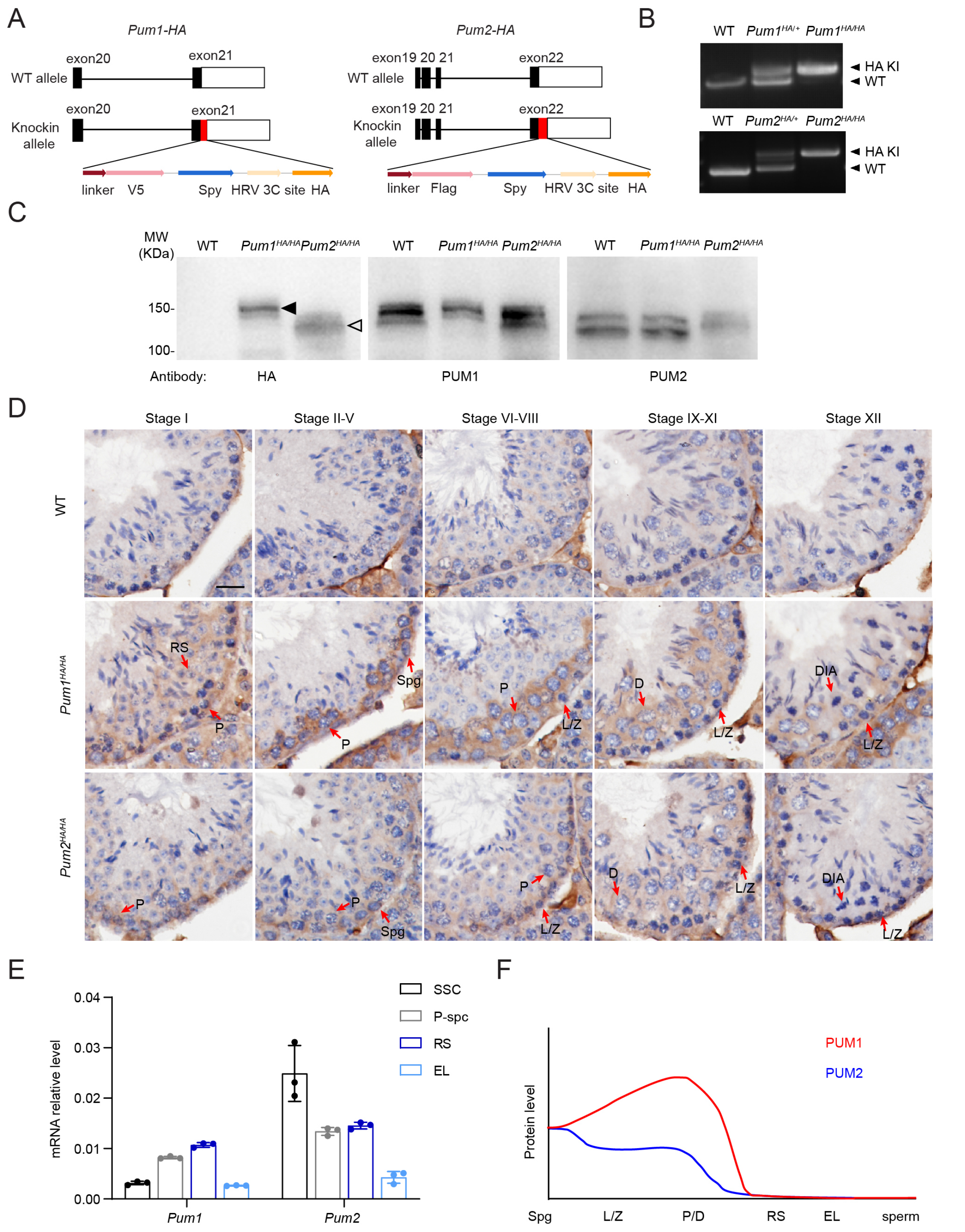

Figure S1. Generation and validation of *Pum1-HA* and *Pum2-HA* knock-in mice. Related to Figure 1 and 2.

(A-C) Targeting strategy, genotyping, and validation of HA-tagged PUM1 and PUM2 knock-in

mice. Schematic of the targeting strategy (A) for generating *Pum1-HA* and *Pum2-HA* alleles. Representative genotyping PCR (B) and Western blot (C) of testis lysates from WT and knock-in mice using anti-HA antibody confirm the expression of PUM1-HA (black arrow) and PUM2-HA (white arrow) fusion proteins.

(D) Characterization of PUM1 and PUM2 expression patterns during spermatogenesis. HA-based immunohistochemistry on $Pum1^{HA/HA}$ and $Pum2^{HA/HA}$ testis sections shows expression across germ-cell stages: spermatogonia (Spg), leptotene/zygotene (L/Z), pachytene (P), diplotene (D), diakinesis (DIA), and round spermatids (RS). Scale bar: 20 μm.

(E) RT-qPCR analysis of *Pum1* and *Pum2* mRNA expression in isolated germ cell populations. Data are presented as mean ± SD.

(F) Schematic summary of PUM1 and PUM2 protein expression dynamics during spermatogenesis based on immunoblot of purified spermatogenic cells and immunohistochemistry of the testis.

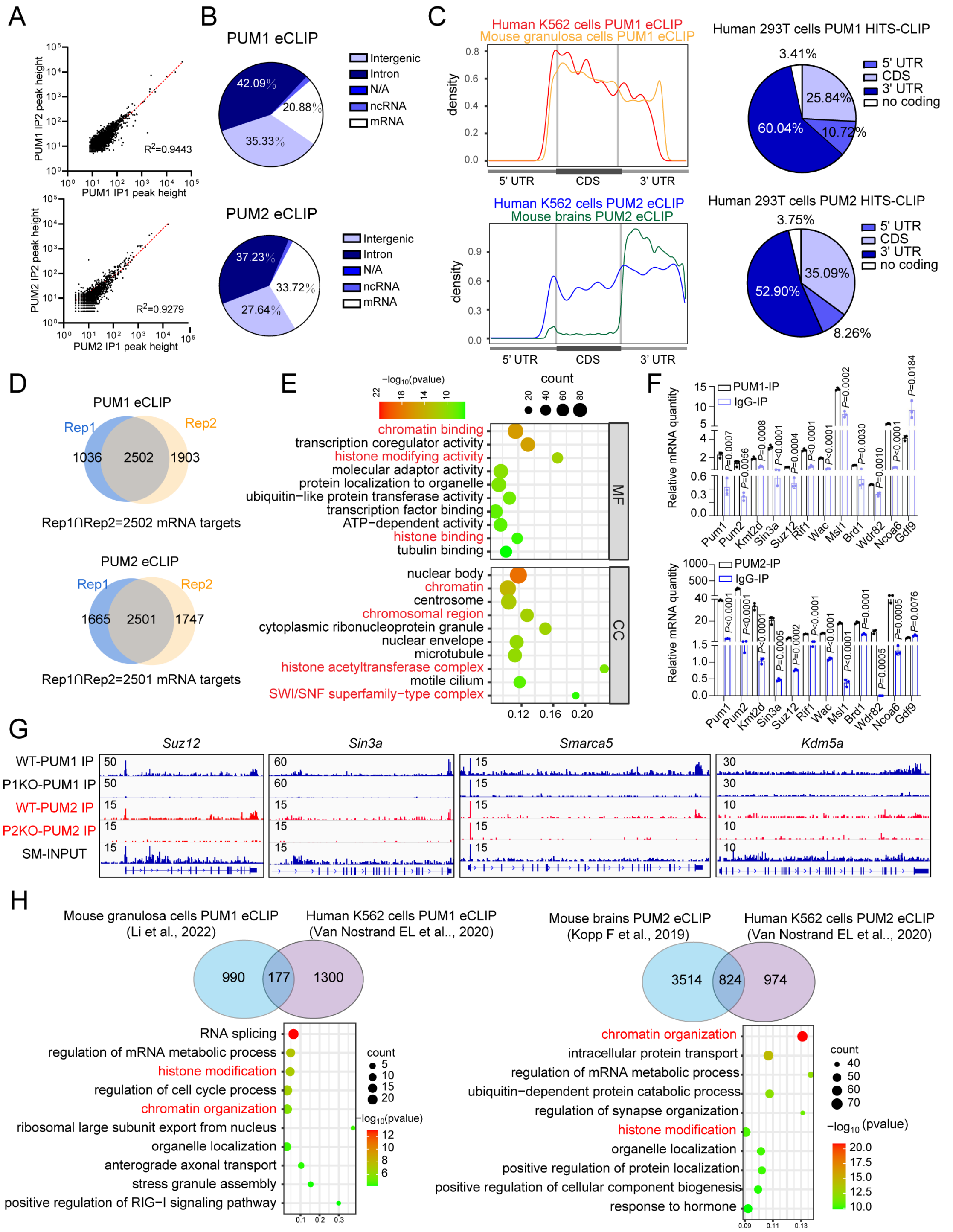

Figure S2. eCLIP reproducibility and validation of PUM1/2 RNA targets. Related to Figure 2.

(A-B) Reproducibility and binding distribution of PUM1/2 eCLIP datasets. Correlation analysis (A) of peak intensities between biological replicates. Genomic distribution (B) of high-confidence peaks for PUM1 and PUM2 eCLIP.

(C) Analysis of PUM1/2 binding patterns across mouse and human cells. Comparison of

genomic feature distributions (e.g., 3′UTR, CDS) for PUM1/2 targets identified in mouse granulosa cells and human K562 cells reveals highly similar binding landscapes.

(D) Overlap of high-confidence targets between PUM1 and PUM2 eCLIP replicates. Venn diagram illustrates the consistency of target identification across independent biological replicates for PUM1 and PUM2 eCLIP.

(E) GO analysis of mRNAs co-bound by PUM1 and PUM2 shows significant enrichment for chromatin-binding (Molecular Function) and chromatin-organization (Cellular Component) functions.

(F) RNA immunoprecipitation (RIP) validates PUM1/2 binding to chromatin-regulator mRNAs. RIP followed by RT-qPCR confirms enrichment of target mRNAs in PUM1/2 immunoprecipitates compared to IgG control. Data are presented as mean ± SD (Student's t-test).

(G) Genome browser visualization of PUM1/2 binding on representative target transcripts. IGV browser tracks display read coverage over full-length transcripts of *Suz12*, *Sin3a*, *Smarca5*, and *Kdm5a*, highlighting gene-body-spanning peaks in both PUM1 and PUM2 eCLIP datasets.

(H) Analysis of mRNAs bound by PUM1 or PUM2 in human and mouse somatic cells identifies chromatin organization and histone modification as the top co-enriched biological processes.

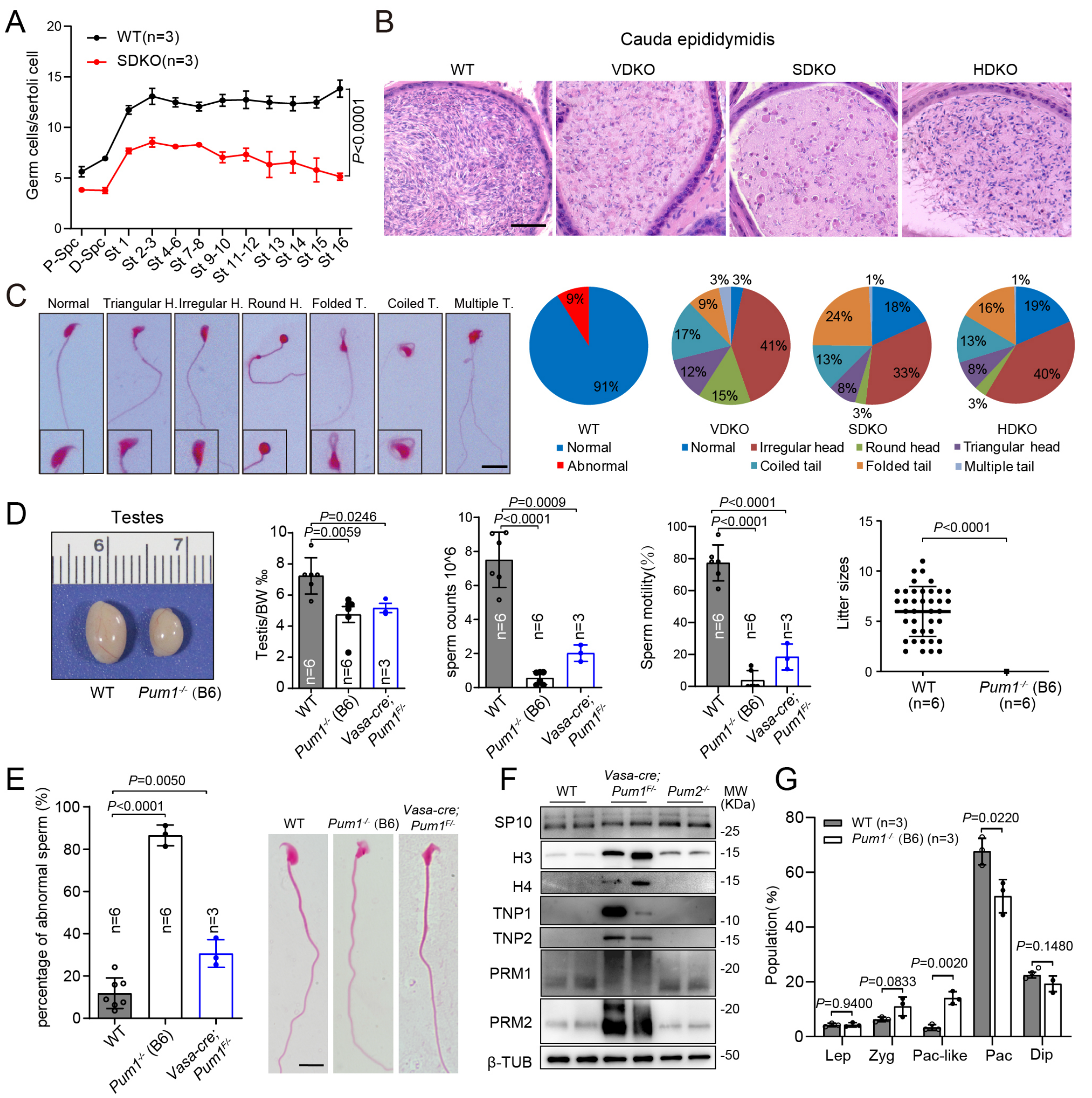

Figure S3. Spermiogenesis defects in SDKO and *Pum1* single-KO mice. Related to Figure 2.

(A) Impaired sperm production across spermiogenic stages in SDKO mice. Quantification of germ cells at key developmental stages - from spermatocytes through spermatozoa - reveals a progressive loss of sperm count in SDKO compared to control mice. Data are presented as mean ± SD (Student's t-test).

(B) Hematoxylin and eosin (H&E) staining of caudal epididymal sections from WT and SDKO mice reveals disrupted sperm accumulation in SDKO. Scale bar: 50 μm.

(C) Increased frequency of abnormal sperm morphology in SDKO mice. Quantification of epididymal sperm showing head deformities, cytoplasmic droplets, or other malformations. Scale bar in representative images: 20 μm.

(D) Complete male sterility in *Pum1* single-KO mice on a C57BL/6 background. Fertility test shows that no pups were produced from matings with systemic *Pum1-KO* males compared to wild-type controls. The germ cell-specific knockout model (*Vasa-cre; Pum1*$^{f/-}$) was also examined for sperm parameters.

(E) Morphological analysis of epididymal sperm from *Pum1-KO* mice..

(F) Expression of histone-to-protamine transition proteins in epididymal sperm from *Pum1-cKO* and *Pum2*$^{-/-}$ mice. Western blot analysis of histones (H3, H4), transition proteins (TNP1, TNP2), and protamines (PRM1, PRM2) shows that *Pum1* deletion specifically disrupts histone-protamine replacement, whereas *Pum2* loss has no significant effect. β-tubulin serves as a loading control.

(G) Altered meiotic progression in C57BL/6 background *Pum1*$^{-/-}$ testes. Chromatin spread analysis and quantification of the relative proportions of meiotic substages (leptotene, zygotene, pachytene, diplotene) from control and *Pum1*$^{-/-}$ mice.

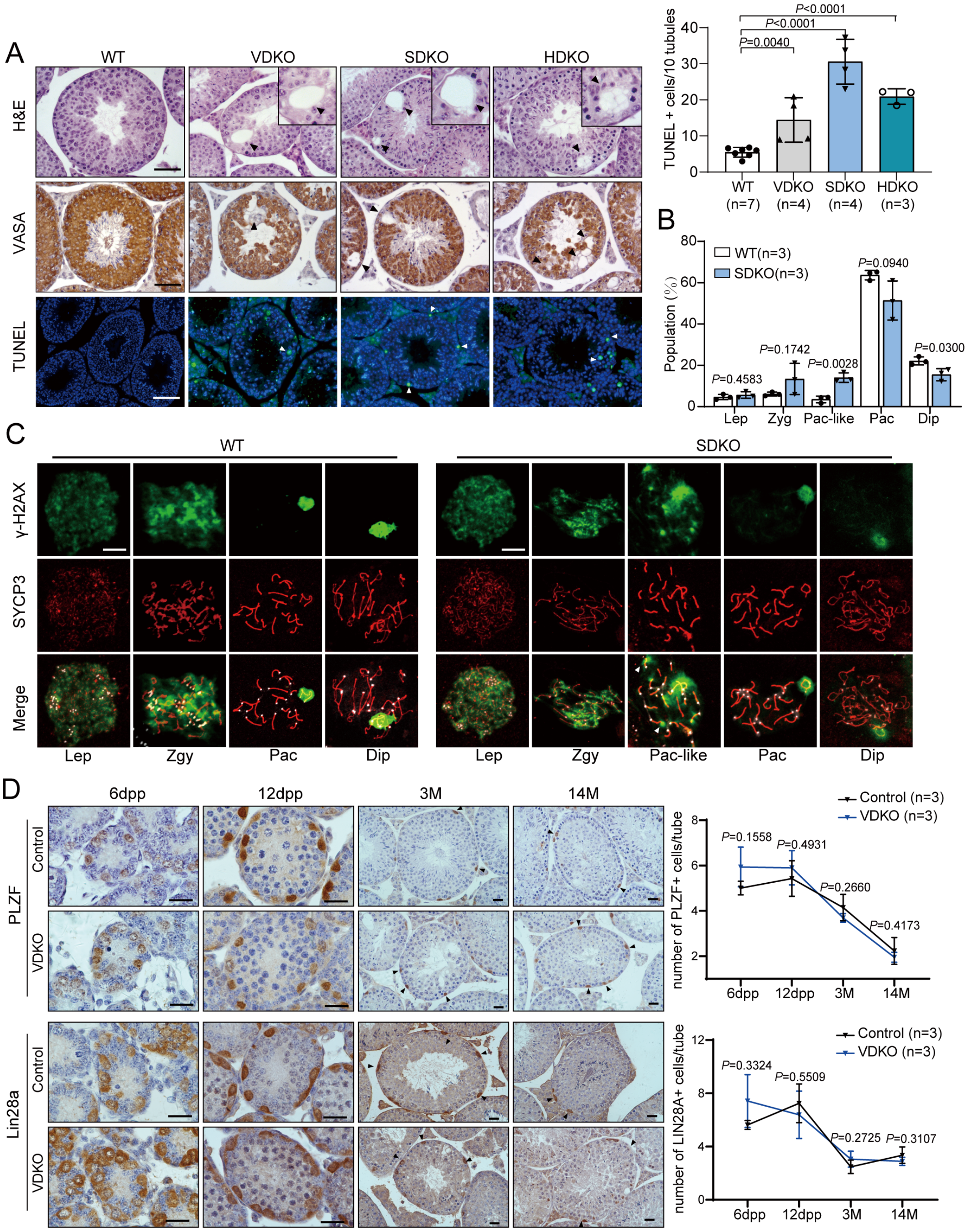

Figure S4. Meiotic defects and SSC maintenance in Pum1/2 cDKO testes. Related to Figure 2.

(A) Germ cell loss and apoptosis in testes of three cDKO models. Hematoxylin and eosin (H&E) staining, VASA immunohistochemistry (IHC), and TUNEL assays of testis sections from control and cDKO mice. Black arrows indicate regions of germ cell loss; white arrows mark TUNEL-

positive apoptotic cells. Scale bar: 20 μm. Apoptotic cells were quantified and analyzed statistically.

(B-C) Altered meiotic progression and modest DNA damage in SDKO spermatocytes. Quantification of meiotic substage distribution reveals increased pachytene-like spermatocytes (B) and slightly delayed zygotene-to-pachytene transition (C) in SDKO testes. Representative images of meiotic chromosomes from control and SDKO spermatocytes highlight γH2AX foci (green) and synaptonemal complex (SYCP3, red). DNA is counterstained with DAPI (blue). Scale bar: 10 μm.

(D) Sustained spermatogonial stem cell (SSC) pool from neonatal to aged VDKO testes. Immunostaining for SSC markers PLZF and LIN28A in testis sections from 6 dpp to 14-month-old control and VDKO mice confirms preserved SSCs despite meiotic and spermiogenic defects. Scale bar: 20 μm. Quantification of PLZF- or LIN28A-positive cells per tubule is shown. Data are presented as mean ± SD (Student's t-test).

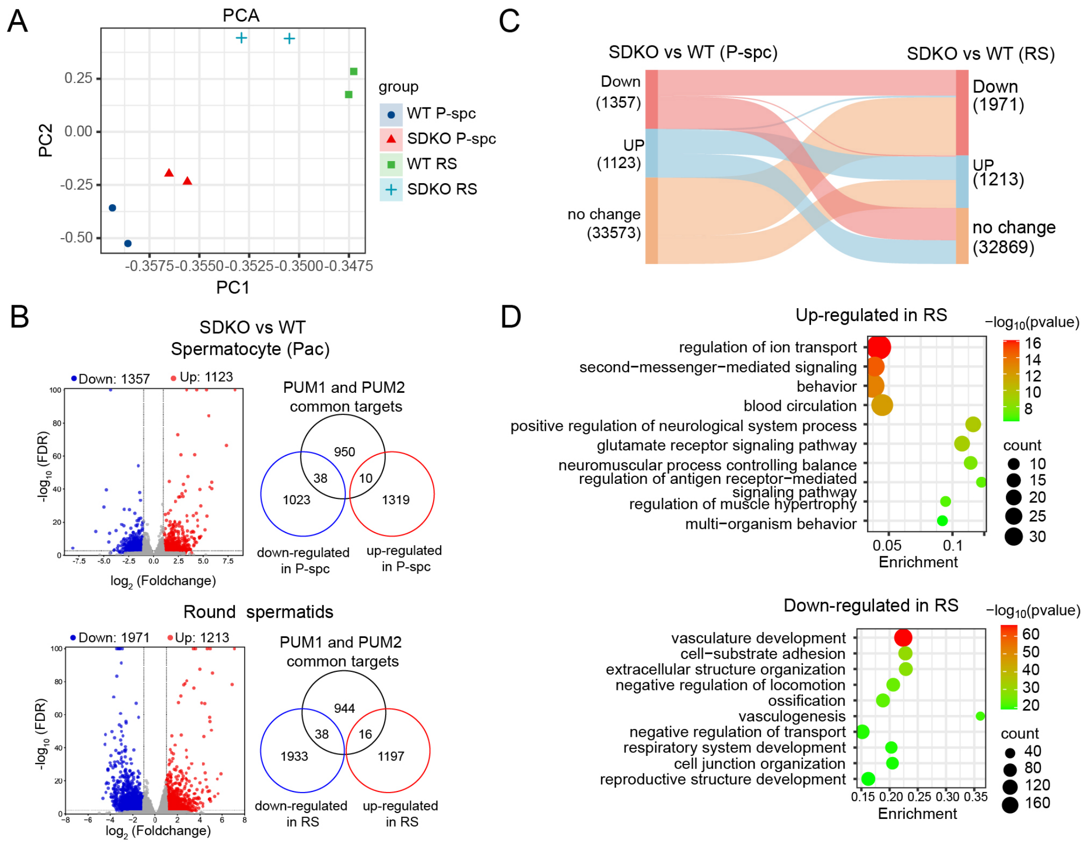

Figure S5. Transcriptome changes in SDKO spermatogenic cells. Related to Figure 3 and Figure 4.

(A) Principal component analysis (PCA) of RNA-seq samples. PCA plot demonstrates distinct clustering of the four experimental groups (wild-type and SDKO spermatocytes; wild-type and SDKO round spermatids).

(B) Widespread transcriptomic alterations in SDKO germ cells. Volcano plots show differentially expressed genes in SDKO versus WT pachytene spermatocytes and round spermatids. In both cell types, only a minor fraction of dysregulated transcripts corresponds to direct PUM1/2 eCLIP targets, as indicated by Venn diagrams.

(C) Sankey diagram illustrating dynamic mRNA expression changes from spermatocytes to round spermatids in SDKO compared to WT.

(D) GO enrichment analysis (Biological Process) of differentially expressed genes in SDKO versus WT round spermatids.

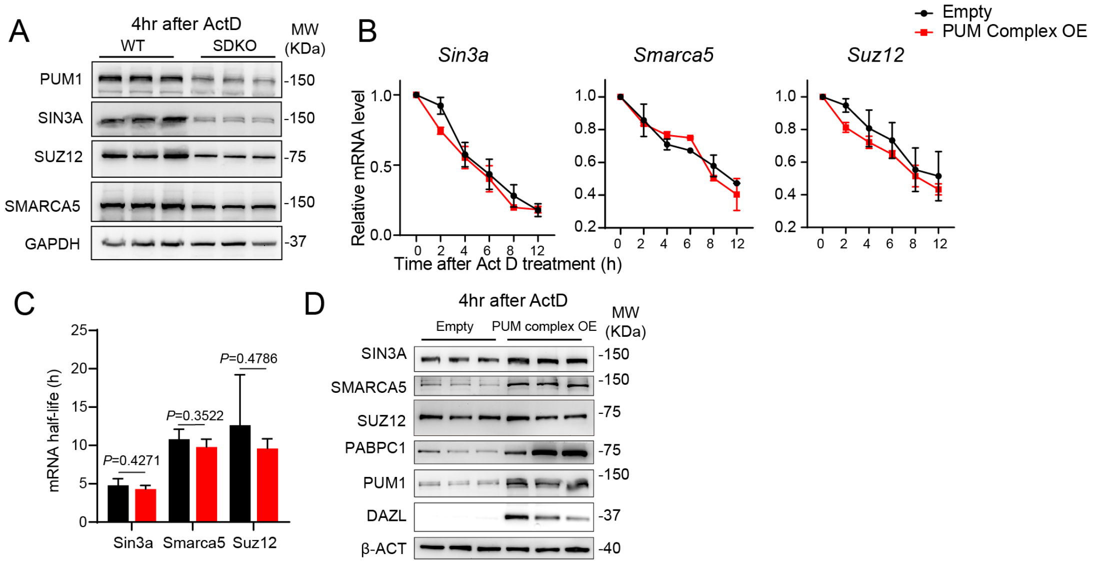

Figure S6. Validation of RNA stability and translational output upon PUM1/2 loss or complex reconstitution. Related to Figs. 4-5

(A) Western blot analysis of SIN3A, SUZ12, and SMARCA5 protein levels in cultured seminiferous tubules from WT and SDKO testes 4 h following Actinomycin D treatment. GAPDH serves as a loading control (see quantification in Fig. 4E).

(B) mRNA decay kinetics of luciferase reporter transcripts in HEK293T cells co-transfected with PUM1, PUM2, DAZL, and PABPC1 expression constructs. Following actinomycin D treatment, reporter mRNA levels were measured over time and normalized to time zero. Exponential regression analysis revealed no significant difference in mRNA half-life upon overexpression of the PUM1/2–DAZL–PABPC1 complex. Increased luciferase activity observed under these conditions therefore reflects enhanced translational output rather than altered mRNA stability. Data represent mean ± SD from three independent experiments.

(C) *Sin3a, Suz12*, and *Smarca5* mRNA levels were measured by RT-qPCR; half-lives ($t_{1/2}$) were calculated using 18S rRNA for normalization.

(D) Western blot of SIN3A and SMARCA5 protein levels 4 hr following Act D treatment shows enhanced expression only when PUM1/2, DAZL, and PABPC1 are co-expressed. β-actin serves as a loading control.

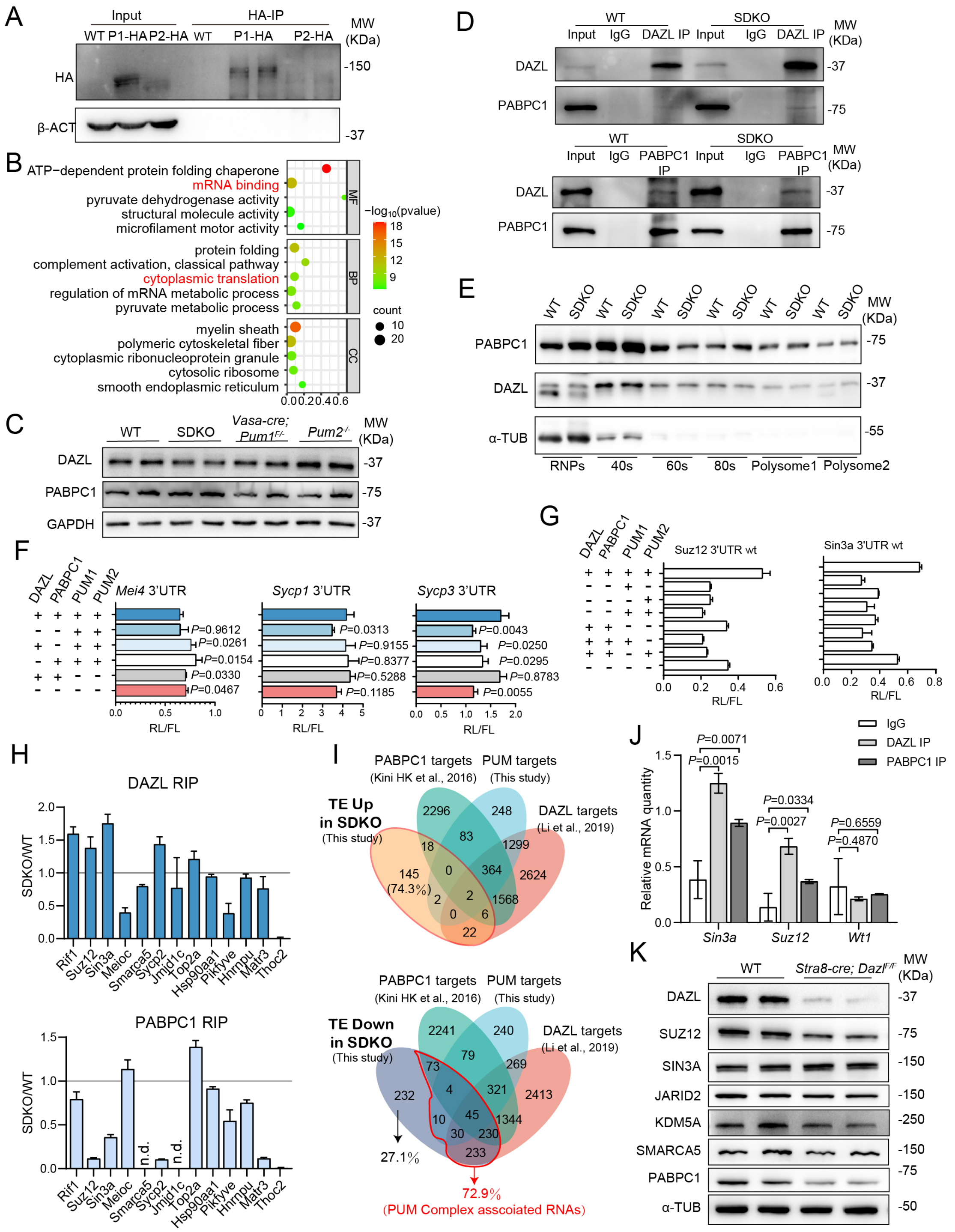

Figure S7. DAZL and PABPC1 expression and interactions are maintained in SDKO testes. Related to Figure 5.

(A) Immunoblot validation of HA-tagged PUM1 and PUM2 immunoprecipitation for mass spectrometry (IP-MS). Western blot analysis of HA-immunoprecipitates from *Pum1$^{HA/HA}$* and *Pum2$^{HA/HA}$* testes lysates confirms efficient purification for subsequent MS analysis.

(B) Gene Ontology enrichment analysis of PUM1/2-interacting proteins highlights significant

enrichment of translation-related functions.

(C) Western blot analysis shows DAZL and PABPC1 protein expression remains similar in SDKO and control testes, with GAPDH as a loading control.

(D) Co-IP indicates DAZL–PABPC1 association is maintained in SDKO testes.

(E) Polysome association of DAZL and PABPC1 is unaltered in SDKO testes. Sucrose-gradient fractionation and Western blot reveal similar polysome-binding profiles for DAZL and PABPC1 in control and SDKO testes.

(F) Dual-luciferase assays containing 3′UTRs of non-target genes (*Mei4*, *Sycp3*) or a PUM1/2 target with unchanged TE (*Sycp1*) show no response to PUM1/2 co-expression, serving as a negative control that supports the specificity of the regulatory mechanism shown in Figure 5D.

(G) PUM1 or PUM2 alone is insufficient for activation: neither paralog individually activates target 3′UTR reporters even with DAZL and PABPC1 present.

(H) RIP-qPCR analysis of DAZL and PABPC1 association with selected target mRNAs in WT and SDKO testes. Enrichment of several transcripts is reduced in PABPC1 immunoprecipitates from SDKO lysates, whereas DAZL association is largely preserved. N.D., not detected. Data are mean ± SD; Student's t-test.

(I) Overlap of PUM1/2-independent but TE-altered targets with published DAZL and PABPC1 CLIP datasets.

(J) RIP-qPCR analysis showing enrichment of *Sin3a* and *Suz12* mRNAs in DAZL and PABPC1 immunoprecipitates from adult mouse testes. *Wt1* was used as a negative control. Statistical significance was determined by Student's t test.

(K) Western blot detection of protein levels for PUM-binding/TE-down targets in DAZL-cKO testes. Analysis of testis lysates from 3-week-old control and DAZL-cKO mice shows that only the chromatin regulator SUZ12 exhibits a slight but consistent decrease.

Supplemental Tables

Table S1. Key Resources Table
Table S2. eCLIP data of mouse testes PUM1 eCLIP and PUM2 eCLIP. Related to Figure 1
Table S3. RNASeq expressionSDKO_WT_SpcRd_Figure 3.
Table S4. Ribo-Seq_RNA-seq_WT_SDKO_Figure 3.
Table S5. OverlapTEdown_RNAdownFigure 3.
Table S6. OverlapPUM1_2Target_TE changed_Figure 3.
Table S7. PUMTargets_HistonePathwayFigure 1_4.
Table S8. MassSpecPUM1_2InteractingProteinsFigure 5.
Table S9. List of primers for PCR and real-time-qPCR, related to STAR Methods.
Table S10. Data related to Figure 4-5 and Figure S6-7.
